# Prediction of the aquatic toxicity of aromatic compounds to tetrahymena pyriformis through support vector regression

**DOI:** 10.18632/oncotarget.17210

**Published:** 2017-04-13

**Authors:** Qiang Su, Wencong Lu, Dongshu Du, Fuxue Chen, Bing Niu, Kuo-Chen Chou

**Affiliations:** ^1^ College of Life Science, Shanghai University, Shanghai 200444, China; ^2^ Department of Chemistry, College of Sciences, Shanghai University, Shanghai 200444, China; ^3^ Department of Life Science, Heze University, Shandong 274500, China; ^4^ Gordon Life Science Institute, Boston, MA 02478, USA; ^5^ Center for Informational Biology, University of Electronic Science and Technology of China, Chengdu 610054, China; ^6^ Center of Excellence in Genomic Medicine Research, King Abdulaziz University, Jeddah 21589, Saudi Arabia

**Keywords:** aromatic compounds, tetrahymena pyriformis, QSAR, genetic algorithm, mRMR

## Abstract

Toxicity evaluation is an extremely important process during drug development. It is usually initiated by experiments on animals, which is time-consuming and costly. To speed up such a process, a quantitative structure-activity relationship (QSAR) study was performed to develop a computational model for correlating the structures of 581 aromatic compounds with their aquatic toxicity to tetrahymena pyriformis. A set of 68 molecular descriptors derived solely from the structures of the aromatic compounds were calculated based on Gaussian 03, HyperChem 7.5, and TSAR V3.3. A comprehensive feature selection method, minimum Redundancy Maximum Relevance (mRMR)-genetic algorithm (GA)-support vector regression (SVR) method, was applied to select the best descriptor subset in QSAR analysis. The SVR method was employed to model the toxicity potency from a training set of 500 compounds. Five-fold cross-validation method was used to optimize the parameters of SVR model. The new SVR model was tested on an independent dataset of 81 compounds. Both high internal consistent and external predictive rates were obtained, indicating the SVR model is very promising to become an effective tool for fast detecting the toxicity.

## INTRODUCTION

Aromatic compounds are used in many industries and consumer products. Many of them are naturally occurring. Hence, they have become widely distributed in nature. Owing to their prevalence in the environment and their likelihood to often elicit unknown toxic effects, it is important to determine their potential hazard. Experimental determination of the toxicity is time consuming and expensive, and can be carried out only for compounds already synthesized. There is a strong need to develop computational tools that can used to predict toxicity. The information thus obtained would be very useful in prioritizing the targets concerned.

As is well known, many different QSAR (Quantitative Structure-Activity Relationship) models have been developed for drug development (see, e.g., [1–7]. The goal of this study was to develop a new QSAR model that can be used to predict the aquatic toxicity of aromatic compounds to tetrahymena pyriformis.

## RESULTS

### Descriptor selection by mRMR-GA-SVR

To examine the quality of a predictor, we need a metrics to quantitatively measure its accuracy. In the current study, a quantity called RMSE was introduced for such a purpose, as defined by

$$\text{RMSE}=\sqrt{\frac{\sum_{i=1}^{n}{\left(p_{i}-e_{i}\right)}^{2}}{n}}$$ (1)

where *e_i_* and *p_i_* denote, respectively, the measured and predicted values for the *i*-th sample; *n* the total number of the samples in the training dataset. Obviously, the smaller the value of RMSE the better the set of selected descriptors.

Listed in Table 1 are the optimal RMSE values obtained by mRMR-GA-SVR under different types of kernel function. As shown in the table, the RMSE value is smaller when using six-descriptor subset under polynomial kernel function. The selected descriptors for QSAR model are energy of the lowest unoccupied molecular orbital (LUMO), the difference between HOMO and LUMO (ΔE), molecular weight (MW), logarithm of the octanol-water partition coefficient (logP), the number of halogen atoms (N_Hal_), and the number of H-bond donors (N_Hdon_).

**Table 1:** RMSE obtained by mRMR-GA-SVR method

RMSE	Kernel function	Descriptors
0.41	Linear kernel	ΔE, logP, ^2^χ, ^3^χ_c_, ^4^χ_pc_, ^3^χ^v^, ^1^κ_a_, Φ, B, N_Hal_
0.38	Polynomial kernel	LUMO, ΔE, MW, logP, N_Hal_, N_Hdon_
0.38	Gauss (RBF) kernel	LUMO, ΔE, MW, logP, ^1^χ^v^, ^3^χ_c_, ^4^χ_pc_, ^4^χ_pc_^v^, ^1^κ_a_, N_Hdon_

### SVR model and its parameter selection

**Figure 1:**
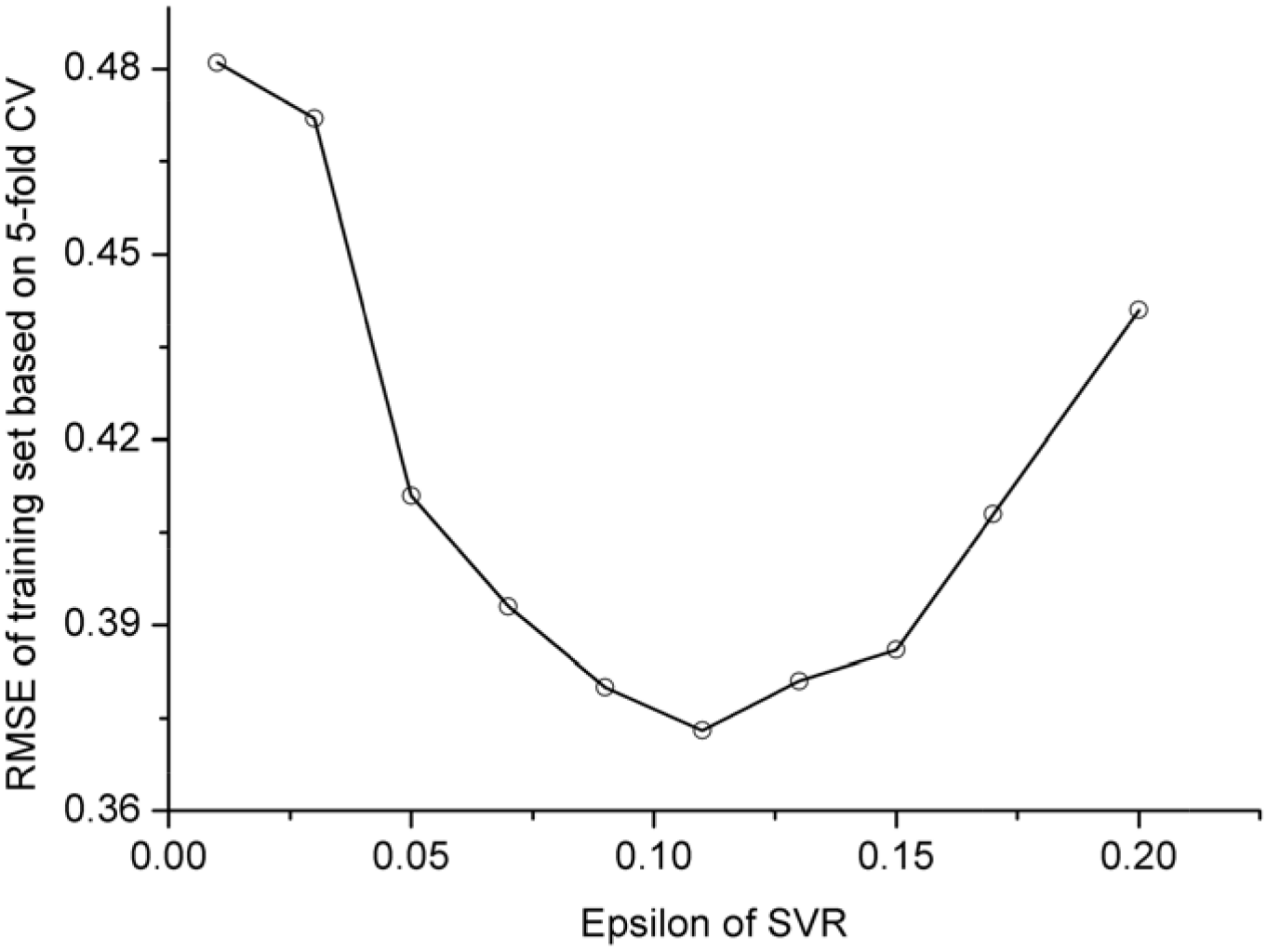
RMSE vs.*ε* in 5-fold CV using polynomial kernel function (C=2.3)

**Figure 2:**
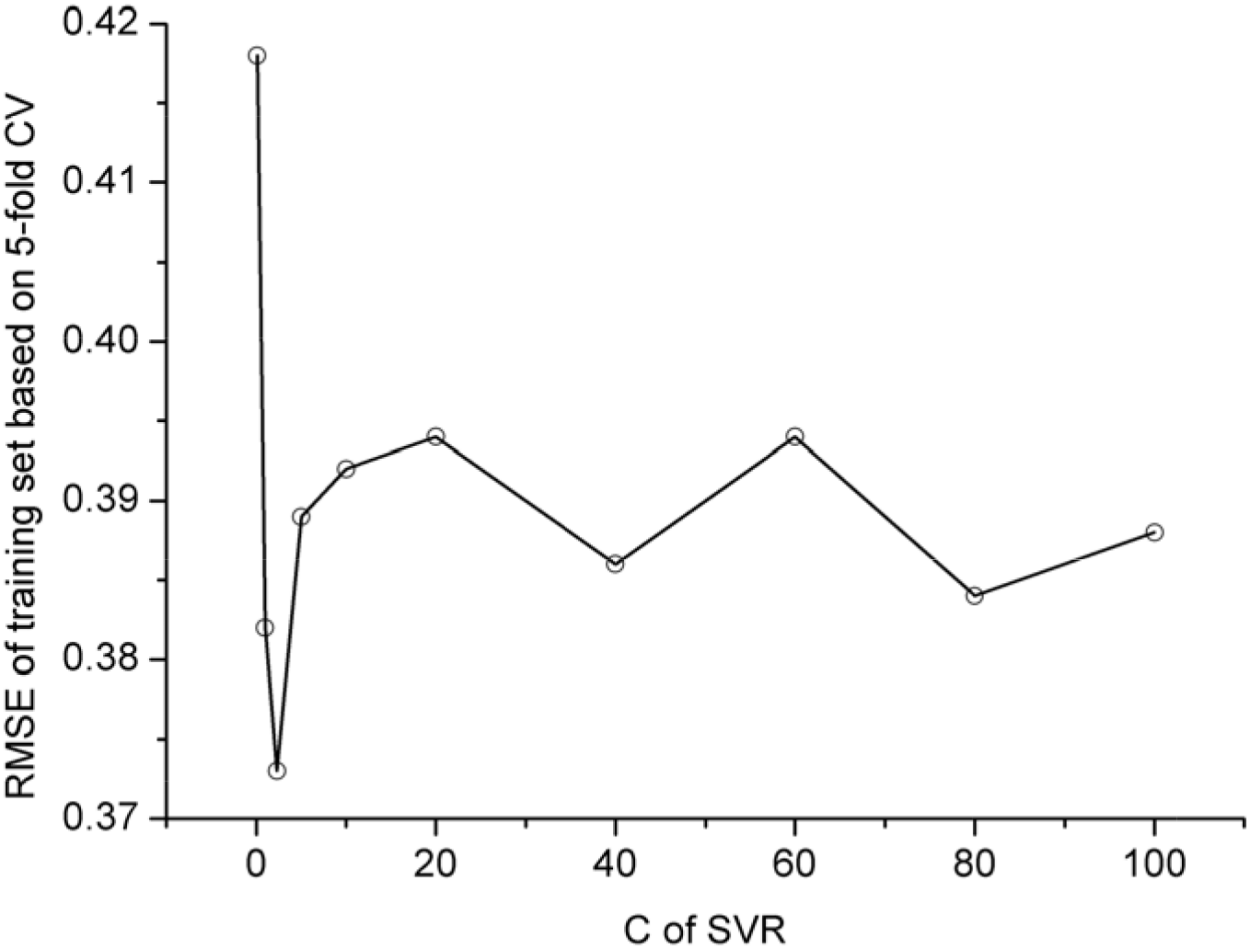
RMSE vs. *C* in 5-fold CV using polynomial kernel function (*ε* =0.11)

In this study, the polynomial kernel function was adopted. The aforementioned SVR model contains two uncertain parameters. One is *C* for the regularization parameter, and the other is ε for the insensitive loss function. Their values were determined by optimizing RMSE (cf. Eq.1) via the 5-fold cross-validation on the training dataset as shown in Figure 1 and Figure 2; i.e.,

C=2.3;    ε=0.11 (2)

Thus, it follows

$$C\cdot \log(IGC_{50}^{-1})={\sum \beta [(x_{i}\cdot x)+1]}^{2}+0.248$$ (3)

where βi =(αi − αi*) is Lagrange coefficient to the corresponding support vector. Listed in Table 2 are the values of RMSE and *R^2^* for log(IGC_50_^−1^) of aromatic compounds obtained by using trained SVR and PLS (partial least squares regression) models. The definition of *R^2^* is given by

$$R^{2}=1-\frac{\sum_{i=1}^{n}{\left(e_{i}-p_{i}\right)}^{2}}{\sum_{i=1}^{n}{\left(e_{i}-\bar{e}\right)}^{2}}$$ (4)

where *^e^_i_* and *p_i_* are the measured and predicted values for the *i*-th sample, e ¯ is the average value of all samples, and *n* is the total number of samples investigated.

**Table 2:** RMSE, *R^2^*, and *Q^2^* for logIGC_50_^−1^ obtained by training set and external test set using different models

Method	Training set			Test set		
	*n*	RMSE	*R^2^*	*l*	RMSE	*Q^2^*
SVR	500	0.38	0.84	81	0.44	0.77
PLS	500	0.42	0.78	81	0.50	0.68
ANN	500	0.40	0.82	81	0.46	0.76

### Validation of the SVR model

**Figure 3:**
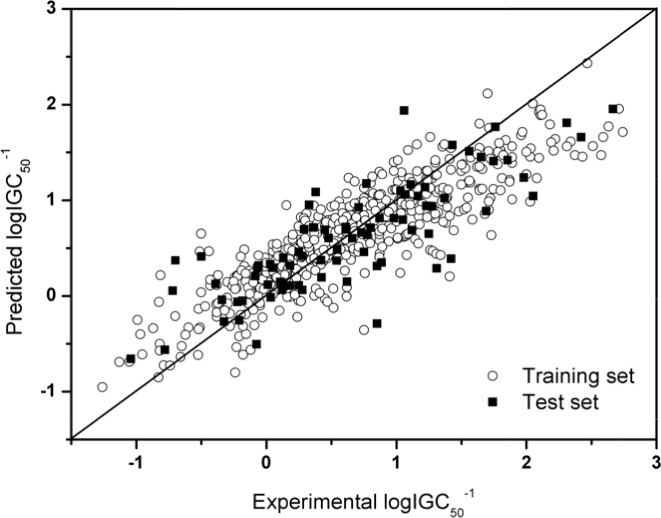
Plot of the experimental vs. predicted logIGC_50_^−1^ values by the SVR model

The model validation was conducted by comparing the predicted and observed logIGC_50_^−1^ of an independent dataset that were not included in the dataset used to train the model. The predictive power of SVR model was evaluated by a quality function Q^2^ as defined by

$$Q^{2}=1-\frac{\sum_{i=1}^{l}{\left(e_{i}-p_{i}\right)}^{2}}{\sum_{i=1}^{l}{\left(e_{i}-\bar{e}\right)}^{2}}$$ (5)

where *l* is the total number of the tested samples, and all the other symbols have the same meanings as in Eq.4. Shown in Figure 3 is a plot of the experimental vs. predicted logIGC_50_^−1^ values by using the SVR model for the training dataset and independent dataset.

## DISCUSSION

### Comparison to the PLS and ANNs

In a benchmark test, the SVR was compared with PLS and ANN (artificial neural network) methods, as shown in Table 2. As shown from the table, the SVR model outperformed both the PLS model and ANN model, indicating that the SVR model would have better generalization ability.

### Effects of the descriptors to the model

The current model was built based on six selected descriptors. To investigate into the effects of the descriptors on the SVR model, let us consider the following outcomes. The quality function Q^2^ was reduced from 0.84 to 0.55 (Table 3) when excluding the MW (molecular weight) descriptor, indicating that molecular weight or volume might have some effects on the toxicity of aromatic compounds.

**Table 3:** RMSE and *Q*^2^, logIGC_50_^−1^ of the training set and external test set of aromatic compounds using different descriptor subsets

Descriptor	Training set		Test set	
	RMSE	*R^2^*	RMSE	*Q^2^*
LUMO, ΔE, MW, logP, N_Hal_, N_Hdon_	0.38	0.84	0.44	0.77
ΔE, MW, logP, N_Hal_, N_Hdon_	0.43	0.82	0.46	0.73
LUMO, MW, logP, N_Hal_, N_Hdon_	0.43	0.82	0.46	0.73
LUMO, ΔE, logP, N_Hal_, N_Hdon_	0.53	0.69	0.66	0.53
LUMO, ΔE, MW, N_Hal_, N_Hdon_	0.55	0.69	0.64	0.56
LUMO, ΔE, MW, logP, N_Hdon_	0.44	0.82	0.47	0.74
LUMO, ΔE, MW, logP, N_Hal_	0.45	0.82	0.46	0.73

### Sensitivity analysis

**Figure 4:**
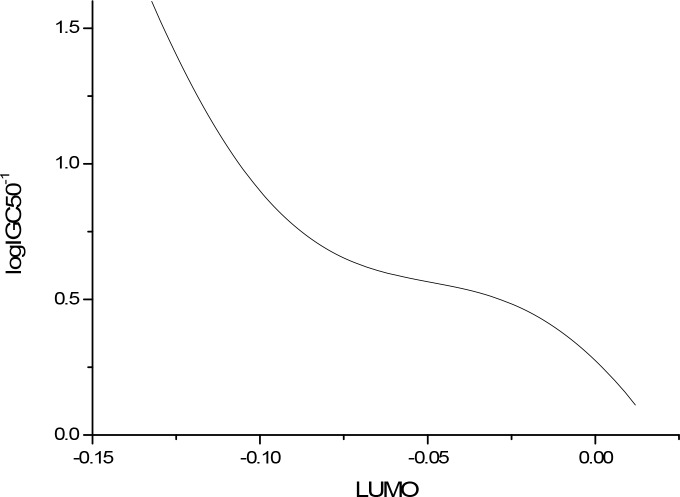
logIGC50^−1^ vs LUMO by SA

**Figure 5:**
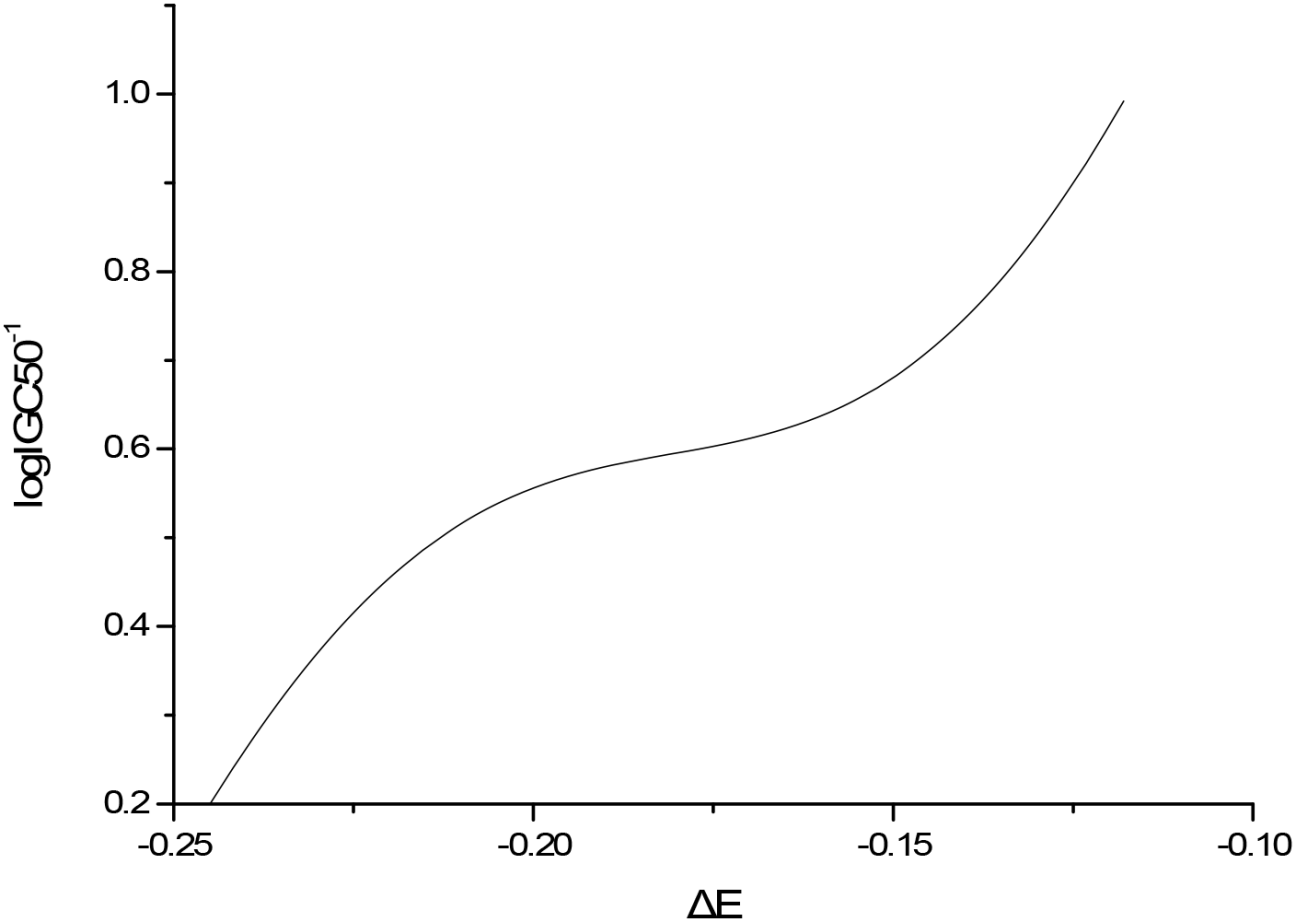
logIGC50^−1^ vs ΔE by SA

**Figure 6:**
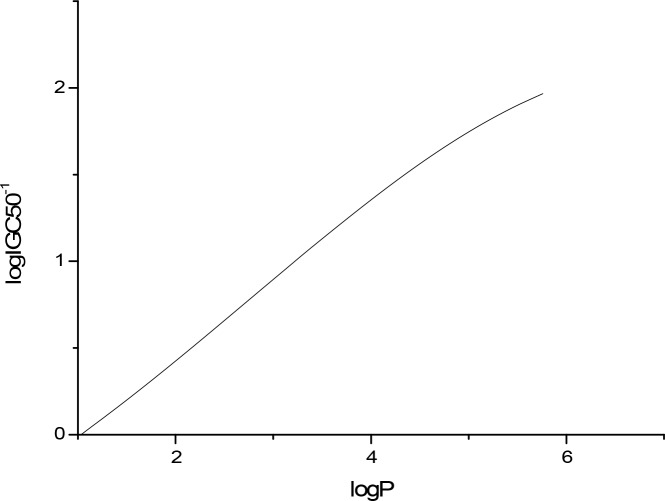
logIGC50^−1^ vs MW by SA

**Figure 7:**
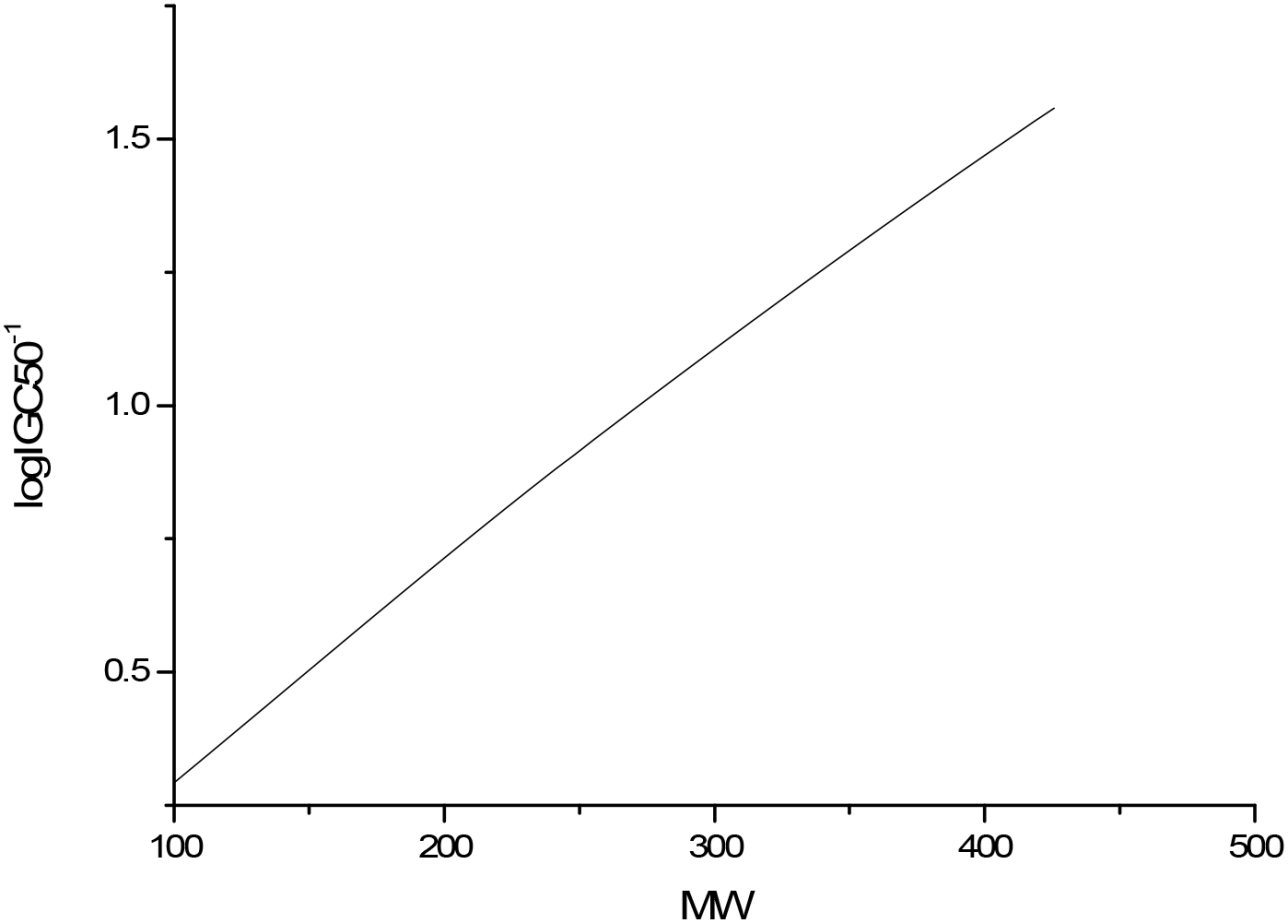
logIGC50^−1^ vs logP by SA

**Figure 8:**
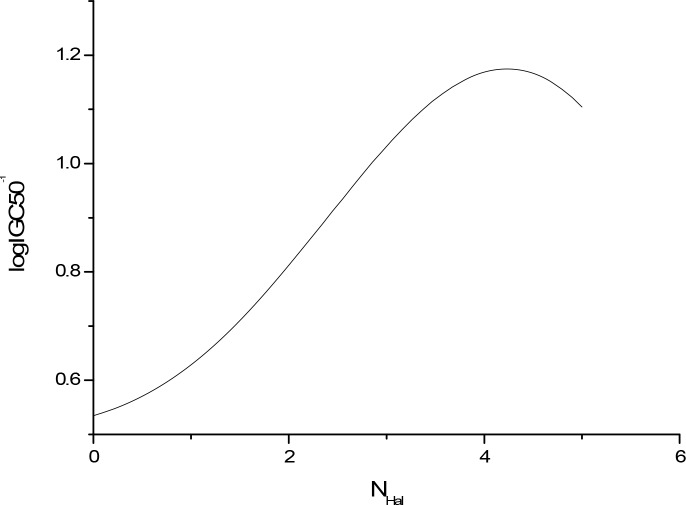
logIGC50^−1^ vs N_Hal_ by SA

**Figure 9:**
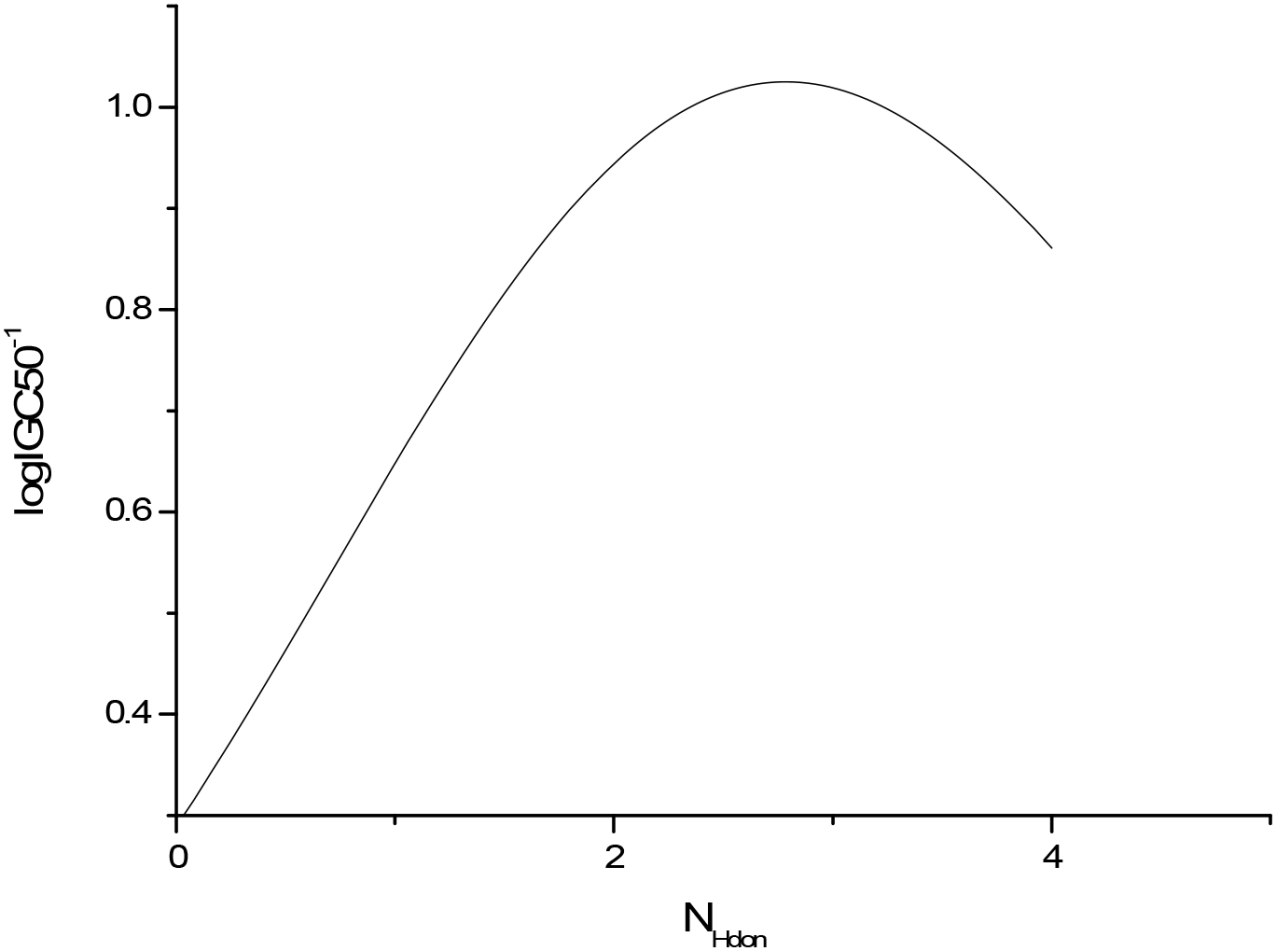
logIGC50^−1^ vs N_Hdon_ by SA

The sensitivity analysis (SA) method was employed to analyze the relationship between attributes and activity. The SA of logP, HOMO and Mass are given in Figures 4-9, respectively. It can be seen from Figures 5-7 that the value of logIGC50^−1^ is increasing with the increment of logP, MW and ΔE. Interestingly, just the opposite trend was observed from Figure 4, where the greater the LUMO is, the lower the logIGC50^−1^ would be, implying that electrons transfer in the process of toxicity interaction is from organic compounds to biological molecules.

### Interpretation of descriptors

The descriptor logP is well known in predictive toxicology. It can describe membrane penetration and interaction with the molecular site of action. The descriptors ΔE and LUMO belong to quantum chemical descriptors. The descriptor ΔE accounts for general stability of a molecule. The descriptor LUMO is related to the relative electrophilicity. In the present study the ab initio Hartree-Fock level calculation provides a strong evidence of toxicity prediction ability of the global and local electrophilicity together with molecular stability. The descriptor N_Hdon_ plays a significant role in solubility behavior. As the hydrogen bond formation increases, water solubility increases (or n-octanol solubility decreases). MW is a general descriptor of size. Halogenated aromatic compounds are related to both the leaving and the electron-withdrawing properties of the group. The toxicity potency is imparted by the leaving ability of the halogen. The addition of the halogen atom increases toxicity in excess of what can be accounted for by hydrophobicity.

## MATERIALS AND METHODS

To establish a really useful statistical predictor, according to the Chou's 5-step rule [8] and realized in a series of recent publications [9–19], we should considered the following five procedures: (1) how to construct or select a valid benchmark dataset to train and test the model; (2) how to represent the samples with an effective mathematical expression that can truly reflect their essential correlation with the target concerned; (3) how to introduce or develop a powerful algorithm to run the prediction; (4) how to properly conduct cross-validation tests to objectively evaluate the anticipated accuracy; (5) how to provide a publically accessible web-server. In the rest of this paper, we are to address these point-by-point.

### Benchmark dataset

In literature, the benchmark dataset usually consists of a training dataset and a testing dataset: the former is constructed for the purpose of training a proposed model, while the latter for the purpose of testing it. As pointed out by a comprehensive review [20], however, there is no need to separate a benchmark dataset into a training dataset and a testing dataset for validating a prediction method if it is tested by the jackknife [21] or subsampling (K-fold) cross-validation because the outcome thus obtained is actually from a combination of many different independent dataset tests.

The benchmark dataset used in this study consists of 581 aromatic compounds with structurally highly heterogeneity and their corresponding toxicity data to the ciliate *tetrahymena pyriformis* in term of log(IGC_50_^−1^) (mmol/l), which means the logarithmic value of the 50% inhibitory growth concentration (IGC_50_) of the ciliates. The toxicity values were taken from the literature [22–29]. A full list of the toxicity values as well as molecular descriptors, or sample formulation [8], used in the SVR model is given in Supporting Information 1. The compounds with the toxicity values ranged from -1.26 to 2.74 log units included a large variety of classes: phenols, anilines, amides, carbonyls, nitro-compounds, cyanides, carboxylic acids, halogenated compounds (F, Cl, Br, I), esters, ethers, pyridines, quinines, and so forth.

The aforementioned 581 chemicals were divided into two sets: one with 500 chemicals used for training the model, and one with 81 chemicals for testing the model. In other words, the benchmark dataset was divided into two subsets: the training dataset and the independent dataset.

The Chemdraw Ultra Version 7.0 (CambridgeSoft Corporation, 2002) software was used for drawing the molecular structures. The molecular structures were optimized by use of the Gaussian 03 [30] (B3LYP/6-311G**) or HyperChem Version 7.5 (HyperCube Inc., 2002) (MM+). Molecular descriptors were obtained by Gaussian 03, HyperChem Version 7.5, and TSAR Version 3.3 (Oxford Molecular Limited, 2000) software's. The mRMR feature pre-selection was performed by using a Red Hat Linux 32-bit-machine version package (http://home.penglab.com/proj/mRMR/). A (procedure for feature selection and a software package containing SVR and PLS methods were programmed in our lab. The validation of the software has been tested in some applications [31–33].

### Molecular descriptors or sample formulation

In developing a powerful statistical predictor, it is very important to represent the statistical samples with an effective formulation that can truly reflect their essential correlation with the target concerned, as done in [34, 35] for proteome systems and in [36–38] for genome systems. Here we are dealing with a compound system, and the corresponding samples should be formulated in a different approach as given below.

The 68 descriptors were calculated with different software and they can be classified into six groups: quantum chemical, electrostatic, topological, geometrical, constitutional, and physicochemical descriptors. A full list of descriptors calculated is provided in Table 4. The methods to calculate descriptors are given below.

**Table 4:** Molecular descriptors and the obtaining methods

Software	Descriptors
Gaussian 03	HOMO energy, LUMO energy, the HOMO-LUMO gap (ΔE), the total molecular energy (E_Tot_), the minimum (Q_Nmax_) and the maximum (Q_Pmax_) atomic partial charge, dipole moment (μ), polarizability (α)
HyperChem release 7.5	Heat of formation (HF), molecular surface area (MSA), molecular volume (MVol), logarithm of the octanol-water partition coefficient (logP), hydration energy (HE), molecular refractivity (MR)
TSAR V3.3	Molecular weight (MW); Kier and Hall simple and valence-corrected molecular connectivity indices (χ); Kappa shape indices (κ); shape flexibility (Φ); Wiener, Randic and Balaban topological indices; E-state indice (S); the number of H-bond donors (N_Hdon_) and acceptors (N_Hacc_); atom counts (oxygen, nitrogen, fluorine, chlorine, bromine, iodine, halogen atoms, heteroatoms); group counts (hydroxyl, amino, aldehyde, nitro, cyano, acid anhydride, methyl)

The 8 quantum chemical descriptors were obtained using Gaussian 03 (shown in Table 4). All the geometries of the aromatic molecules (except I) are minimized at the Hartree-Fock level of theory with the 6-311 G** basis set followed by frequency calculations using the Gaussian 03 package.

The 6 electrostatic descriptors were calculated using the semi-empirical quantum-chemical method PM3 in HyperChem 7.5 software package (listed in Table 4). Geometry optimizations based on molecular mechanics (using the MM+ force field) and semi-empirical quantum mechanical calculations using PM3 were used to find the coordinates of molecular structures that represent a potential energy minimum. For geometry optimization using both molecular mechanics and semi-empirical quantum mechanical calculations, at the final stage of refinement, the Polak-Ribiere routine with RMS gradient of 0.001 kcal Å mol^−1^ as the termination condition was used.

Other descriptors were calculated with TSAR Version 3.3 as noted in Table 4.

### mRMR

The mRMR (minimum redundancy maximum relevance) method [39] selects features that have the highest relevance with the target class and are also minimally redundant, i.e., selects features that are maximally dissimilar to each other. The idea of mRMR has been widely used to analyze various biological sequences (see, e.g., [10, 40–44]). For more information about mRMR, see [39, 40], where a detailed procedure has been elaborated. Hence there is no need to repeat here.

### Descriptor selection for mRMR-GA-SVR

The performance of QSAR model closely depends on how to select the features of molecular structures (Table 5). In this study, a comprehensive feature selection method called mRMR-GA-SVR was introduced as described below.

**Table 5:** Parameters of the GA-SVR feature selection

Parameter	Value	Parameter	Value
Population Size	50	Regression method	SVR
Maximum generations	100	Cross-validation	5-fold
Probability of crossover	0.75	Fitness function	*RMSE*
Probability of mutation	0.01	Regularization parameter (*C*)	10

In the first stage, the mRMR approach was applied for feature selection as done in [45]. There are three distinct advantages by doing so: (1) it can select the features that have better representativity for the targets concerned; (2) it can avoid the high dimension disaster problem [46]; and (3) it can narrow down the search space for the subsequent study.

In the second stage, a GA-based SVR or GA-SVR approach was applied to refines the mRMR-selected-features. The GA algorithm can be found in [47, 48]. The codes for GA-SVR program had been written in our lab using the Visual Basic language.

### SVR algorithm

The Support Vector Machines (SVM) is a machine-learning algorithm, which has been widely used in many areas of bioinformatics (see, e.g., [10, 37, 49–54]). The key idea of SVM is to construct a separating hyper-plane so as to maximize the margin between the positive dataset and negative dataset. For a brief formulation of SVM and how it works, see the papers [55, 56]; for more details about SVM, see a monograph [57]. In SVR, the basic idea is to map the data *X* into a higher-dimensional feature space F via a nonlinear mapping Φ and then to do linear regression in this space. For more details about SVR, see Supporting Information 2.

### Web server

As pointed out in [58], user-friendly and publicly accessible web-servers represent the future direction for developing practically more useful predictors or any computational tools. Actually, user-friendly web-servers as given in a series of recent publications [9, 10, 59–68] will significantly enhance the impacts of theoretical work because they can attract the broad experimental scientists [69]. Once the funding is available for purchasing the needed facilities, we will establish a web-server for the new QSAR model reported in this paper.

## CONCLUSIONS

The SVR approach was used to develop a new QSAR model for predicting logIGC_50_^−1^ for a wide-ranging and heterogeneous set of aromatic compounds. The mRMR-GA-SVR method was applied for descriptor selection. The results have indicated that the mRMR-GA-SVR method is a very effective for QSAR analysis. The prediction ability of SVR was tested by an independent dataset of 81 aromatic compounds. The *R*^2^ for the training set for SVR is 0.84. And the *Q^2^* for the independent test set is 0.77. It is anticipated that SVR will become a useful high throughput tool for detecting the potential toxicity to *Tetrahymena pyriformis* for a diverse set of aromatic compounds.

## SUPPLEMENTARY MATERIALS FIGURES AND TABLES




