# Supplementary material for: Prediction of the aquatic toxicity of aromatic compounds to tetrahymena pyriformis through support vector regression
Source: Oncotarget. 2017 Apr 13;8(30):49359–69. doi: 10.18632/oncotarget.17210 (PMC5564774; doi:10.18632/oncotarget.17210)
Supplement: Supplementary file 1 [file oncotarget-08-49359-s001.pdf]

Prediction of the aquatic toxicity of aromatic compounds to tetrahymena pyriformis through support vector regression

SUPPLEMENTARY MATERIALS

Supporting Information 1

Supporting Information 1: Chemical name, Chemical Abstract Service (CAS) number, experimental and predicted toxicity values (logIGC50-1) to Tetrahymena pyriformis, and calculated descriptors used in SVR model

See Supplementary File 1

Supporting Information 2

SVR algorithm

SVM algorithm are mainly developed by Vapnik and his co-workers. SVM can be applied to regression by the introduction of an alternative loss function and the results appear to be very encouraging. In SVR, the basic idea is to map the data *X* into a higher-dimensional feature space F via a nonlinear mapping Φ and then to do linear regression in this space. Therefore, regression approximation addresses the problem of estimating a function based on a given data set $G = \{(x_i; d_i)\}_{i=1}^{l}$ (x_i is input vector, d_i is the desired value). SVM approximates the function in the following form:

$$y = \sum_{i=1}^{l} w_i \phi_i(x) + b \qquad (1)$$

Where $\{\phi_i(x)\}_{i=1}^{l}$ is the set of mappings of input features, and $\{w_i\}_{i=1}^{l}$ is a vector of weights in the features space, and*b* are coefficients. They are estimated by minimizing the regularized risk function *R(C)*:

$$R(C) = C\frac{1}{N}\sum_{i=1}^{N} L_\varepsilon(d_i, y_i) + \frac{1}{2}\|w\|^2 \qquad (2)$$

Where

$$L_\varepsilon(d, y) = \begin{Bmatrix} |d-y| - \varepsilon \; for \; |d-y| \geq \varepsilon \\ 0 \; otherwise \end{Bmatrix} \qquad (3)$$

And ε is a prescribed parameter in the insensitive loss function.

In Eq. (2), $C\frac{1}{N}\sum_{i=1}^{N} L_\varepsilon(d_i, y_i)$ is the so-called empirical error (risk), which is measured by ε-insensitive loss function $L_\varepsilon(d, y)$, which indicates that it does not penalize errors below ε. The second term, $(1/2)\|w\|^2$ is used as a measurement of function flatness. *C* is a regularization constant determining the tradeoff between the training error and the model flatness. Introduction of slack variables "ζ" leads Eq. (2) to the following constrained function:

$$\text{MaxR}(w, \xi^*) = \frac{1}{2}\|w\|^2 + C^* \sum_{i=1}^{n}(\xi_i + \xi_i^*) \qquad (4)$$

$$\text{s.t.} \; w\phi(x_i) + b - d_i \leq \varepsilon + \xi_i$$
$$d_i - w\phi(x_i) - b \leq \varepsilon + \xi_i^*$$
$$\xi_i, \xi_i^* \geq 0 \qquad (5)$$

Thus, decision function Eq. (1) becomes the following form:

$$f(x, \alpha_i, \alpha_i^*) = \sum_{i=1}^{l}(\alpha_i - \alpha_i^*)K(x_i, x_j) + b \qquad (6)$$

In Eq. (6), α_i, α_i^* are the introduced Lagrange multipliers. They satisfy the equality $\alpha_i \cdot \alpha_i^* = 0, \alpha_i \geq 0, \alpha_i^* \geq 0; i = 1, \cdots, l$, and are obtained by maximizing the dual form of Eq. (4), which has the following form:

$$\phi(\alpha_i, \alpha_i^*) = \sum_{i=1}^{l} d_i(\alpha_i - \alpha_i^*) - \varepsilon\sum_{i=1}^{l}(\alpha_i + \alpha_i^*) - \frac{1}{2}\sum_{i=1}^{l}\sum_{j=1}^{l}(\alpha_i - \alpha_i^*)(\alpha_j - \alpha_j^*)K(x_i, x_j) \qquad (7)$$

with the following constrains:

$$0 \leq \alpha_i \leq C \quad i = 1 \cdots\cdots, l$$
$$0 \leq \alpha_i^* \leq C \quad i = 1 \cdots\cdots, l \qquad (8)$$
$$\sum_{i=1}^{l}(\alpha_i - \alpha_i^*) = 0$$

Solving Eq. (7) with constraints Eq. (8) determines the Lagrange multipliers, α_i, α_i^* Based on the Karush-Kuhn-Tucker (KKT) conditions of quadratic programming, only a number of coefficients $(\alpha_i - \alpha_i^*)$ will assume nonzero values, and the data points associated with them are referred to as support vectors.

In Eq. (6), $K(x_i, x_j)$ is the kernel function. The value is equal to the inner product of two vectors x_i and x_j in the feature space ϕ(x), i.e. $K(x_i, x_j) = \phi(x_i)\,\phi(x_j)$. The elegance of using kernel function lied in the fact that one can deal with feature spaces of arbitrary dimensionality without having to compute the map ϕ(x) explicitly. Any function that satisfies Mercer's condition can be used as the kernel function. Some commonly used forms of kernel functions list as follows:

$$\text{Linear Kernel } K(x_i, x_j) = ((x_i \cdot x_j) + \theta) \qquad (9)$$

Gaussian (RBF) Kernel $K\left(x_i, x_j\right) = \exp(-\gamma^* \| x_i - x_j \|^2)$ (10)

Polynomial Kernel $K\left(x_i, x_j\right) = \left((x_i \cdot x_j) + \theta\right)^d$ (11)

The general steps of SVR algorithm are: (1) Normalize all the data; (2) Set variables C and ε; (3) Structure a quadratic programming (QP) problem Eq.(4); (4) Transfer QP problem into the formula of Lagrange function; (5) Solve QP problem; (6) Obtain the parameter w and b.

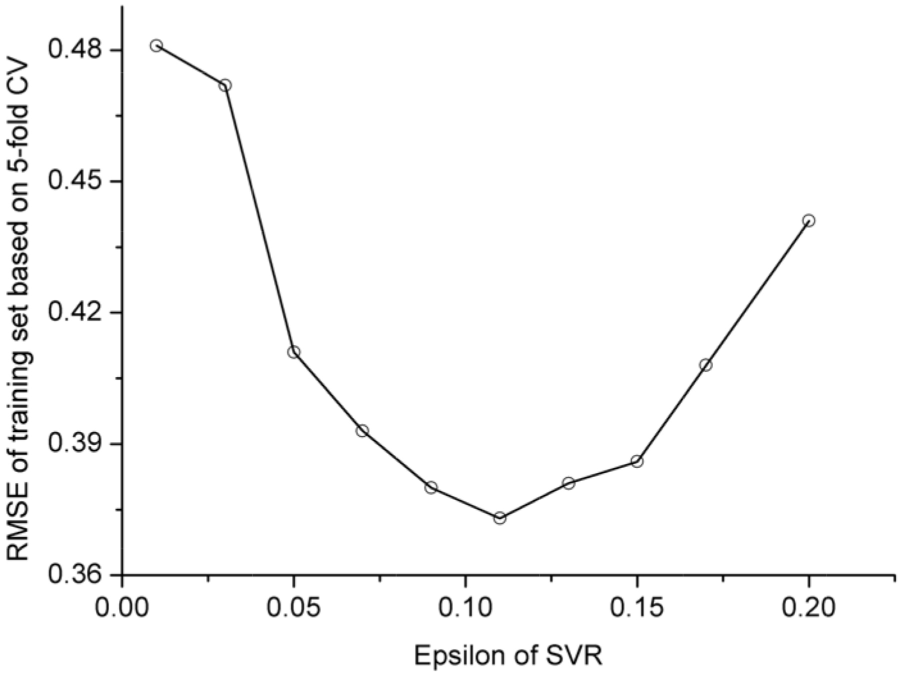

Supplementary Figure 1: *RMSE* vs. ε in 5-fold CV using polynomial kernel function (C=2.3).

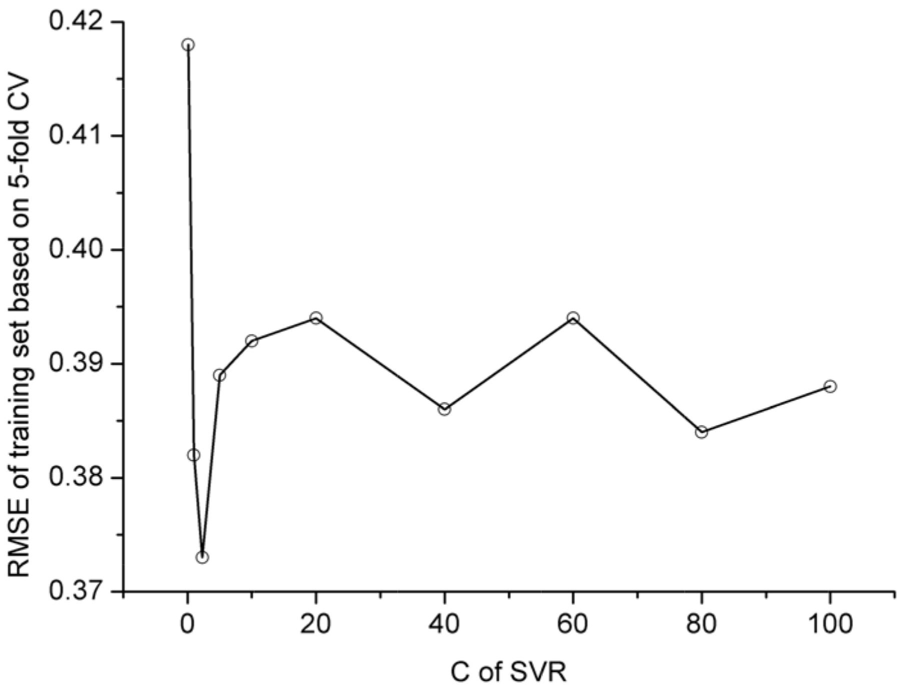

Supplementary Figure 2: ***RMSE*** **vs.** ***C*** **in 5-fold CV using polynomial kernel function (ε =0.11).**

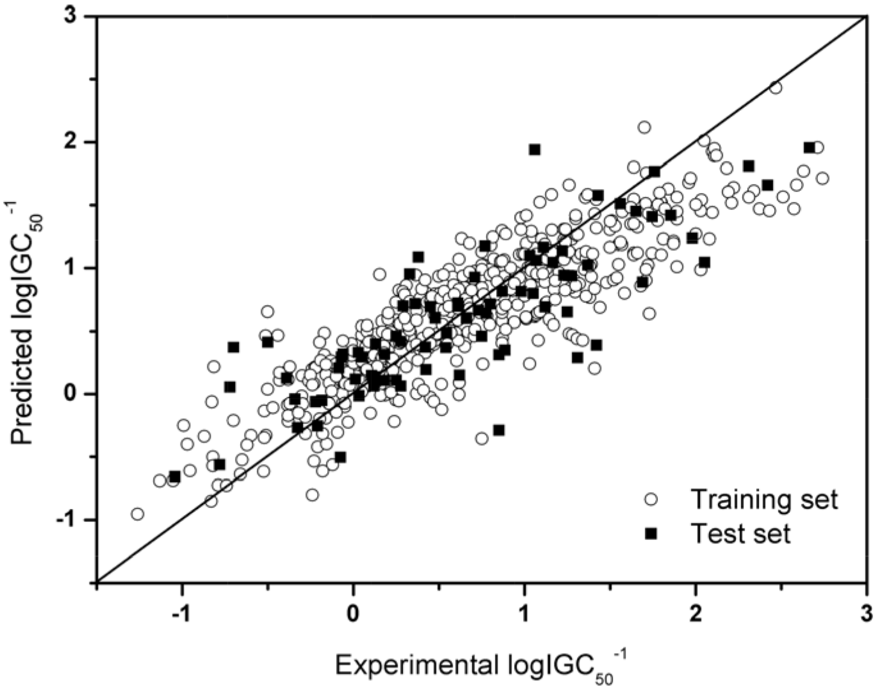

Supplementary Figure 3: Plot of the experimental vs. predicted $logIGC_{50}^{-1}$ values by the SVR model.

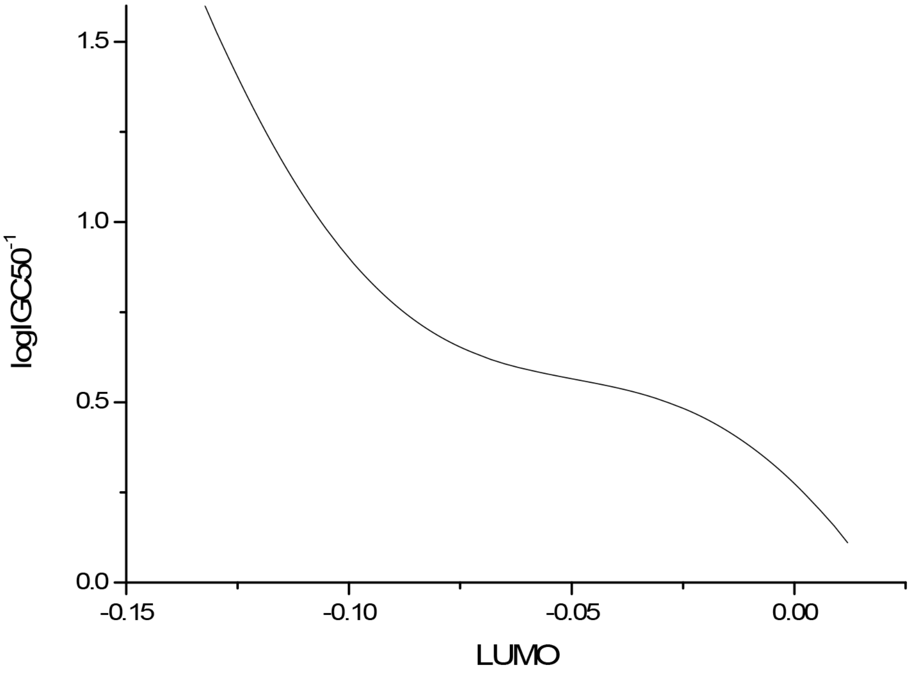

Supplementary Figure 4: logIGC50^{-1} vs LUMO by SA.

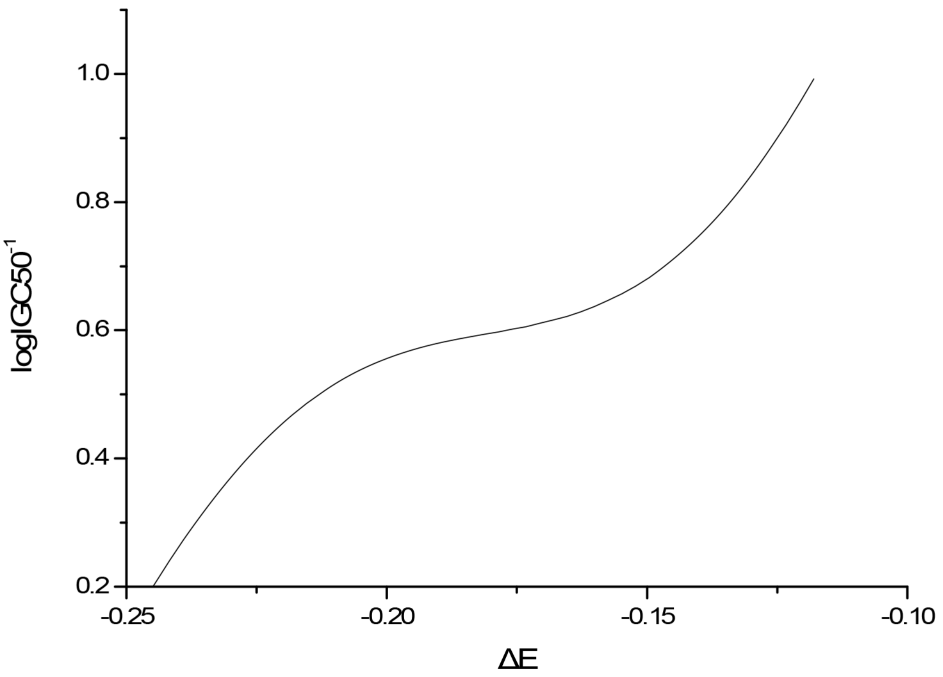

Supplementary Figure 5: logIGC50^{-1} vs ΔE by SA.

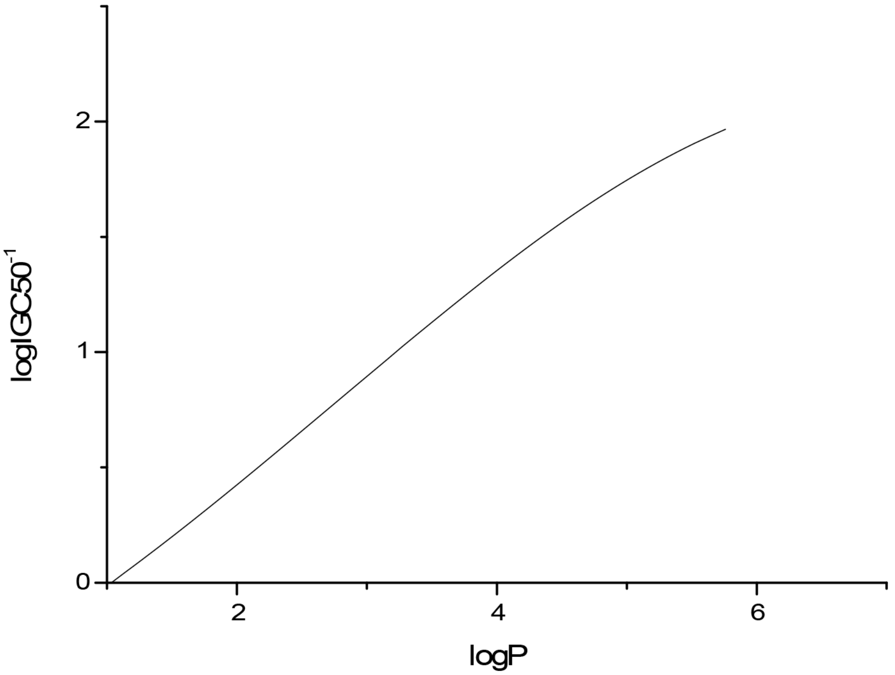

Supplementary Figure 6: logIGC50^{-1} vs MW by SA.

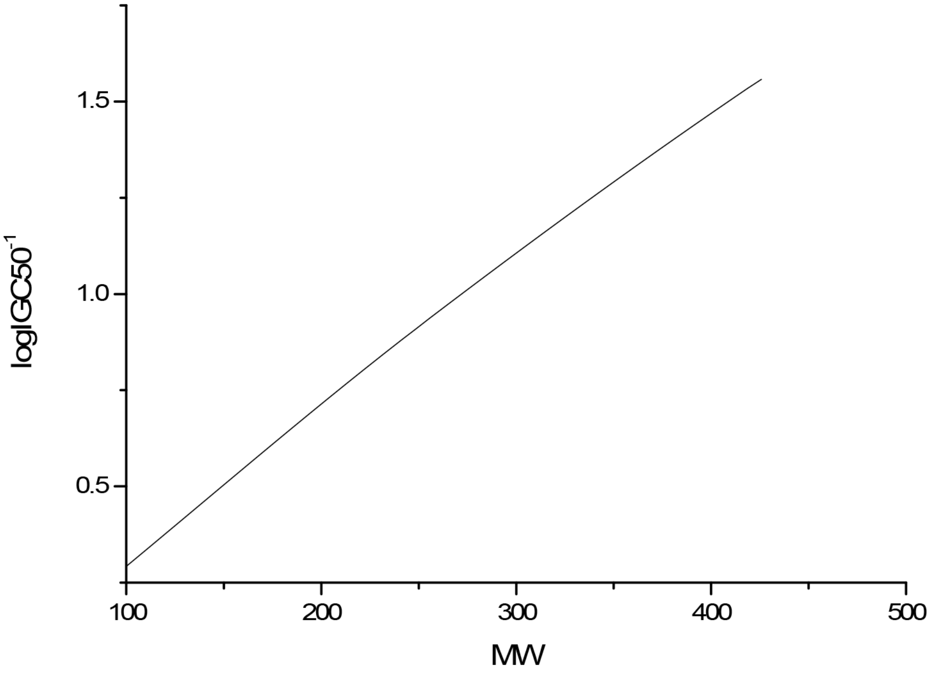

Supplementary Figure 7: logIGC50^{-1} vs logP by SA.

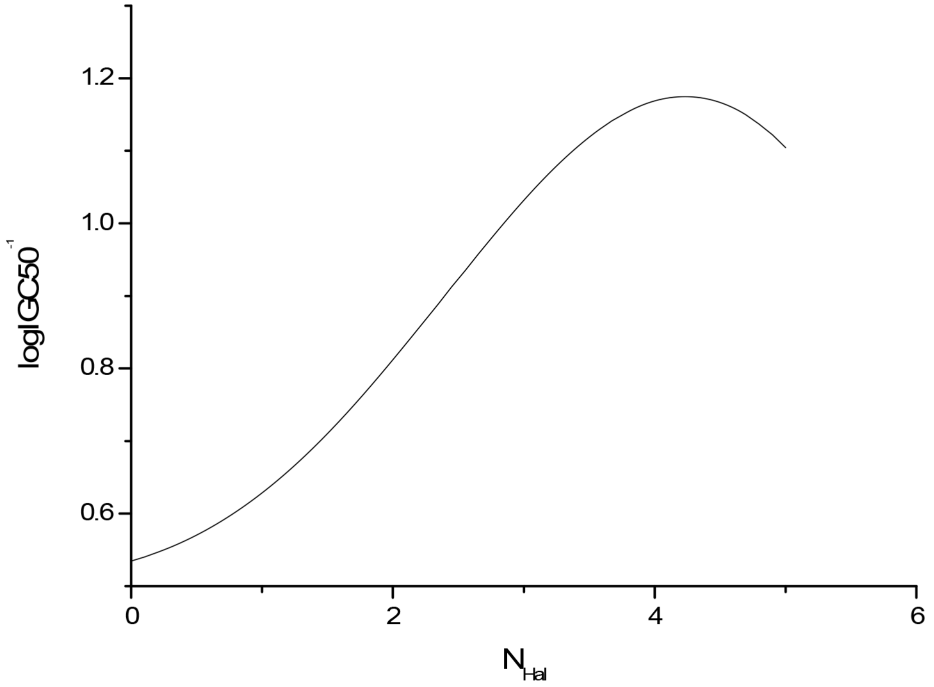

Supplementary Figure 8: logIGC50^{-1} vs N$_{Hal}$ by SA.

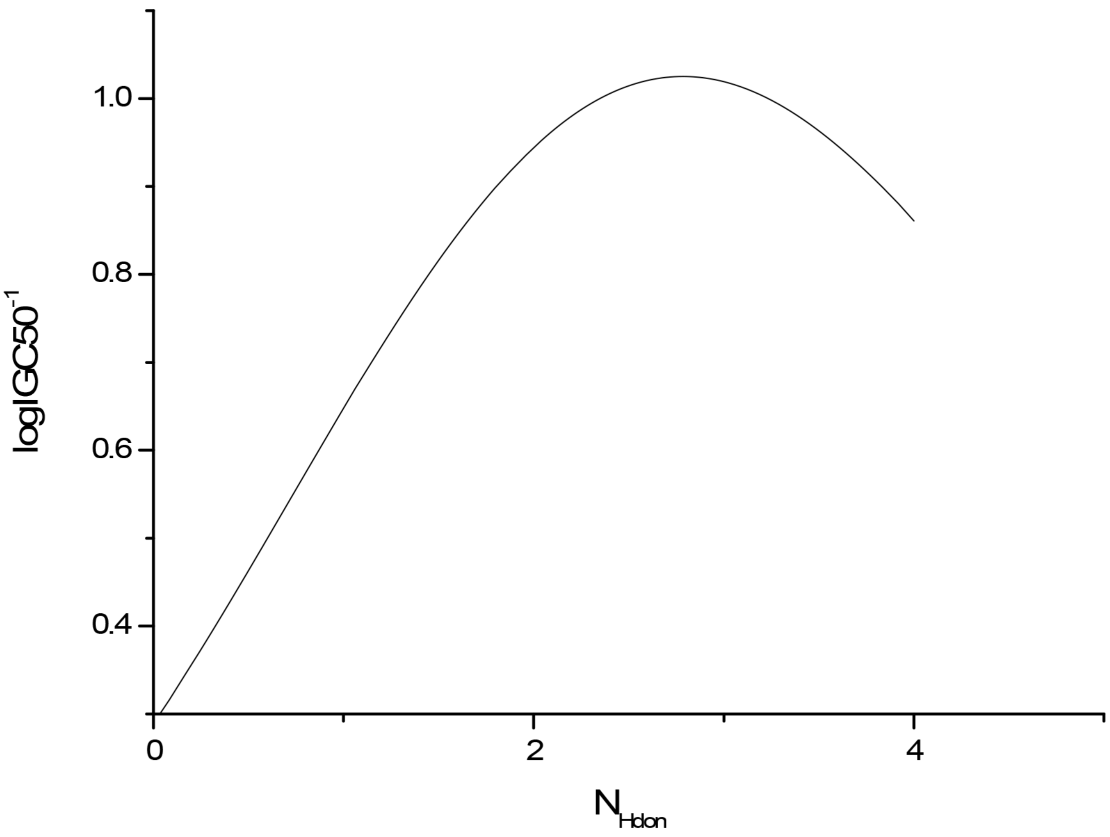

Supplementary Figure 9: $logIGC50^{-1}$ vs N_{Hdon} by SA.

Supplementary Table 1: *RMSE* obtained by mRMR-GA-SVR method

RMSE	Kernel function	Descriptors
0.41	Linear kernel	ΔE, logP, $^{2}\chi$, $^{3}\chi_{c}$, $^{4}\chi_{pc}$, $^{3}\chi^{v}$, $^{1}\kappa_{a}$, Φ, B, N_{Hal}
0.38	Polynomial kernel	LUMO, ΔE, MW, logP, N_{Hal}, N_{Hdon}
0.38	Gauss (RBF) kernel	LUMO, ΔE, MW, logP, $^{1}\chi^{v}$, $^{3}\chi_{c}$, $^{4}\chi_{pc}$, $^{4}\chi_{pc}{}^{v}$, $^{1}\kappa_{a}$, N_{Hdon}

Supplementary Table 2: *RMSE, R^2*, and *q^2* for *$logIGC_{50}^{-1}$* obtained by training set and external test set using different models

Method	Training set			Test set		
	n	*RMSE*	*R^2*	l	*RMSE*	*q^2*
SVR	500	0.38	0.84	81	0.44	0.77
PLS	500	0.42	0.78	81	0.50	0.68
ANN	500	0.40	0.82	81	0.46	0.76

Supplementary Table 3: ***RMSE and q^2 logIGC$_{50}^{-1}$ of the training set and external test set of aromatic compounds using different descriptor subsets***

Descriptor	*Training set*		*Test set*	
	RMSE	*R^2*	*RMSE*	*q^2*
LUMO, ΔE, MW, logP, N_{Hal}, N_{Hdon}	0.38	0.84	0.44	0.77
ΔE, MW, logP, N_{Hal}, N_{Hdon}	0.43	0.82	0.46	0.73
LUMO, MW, logP, N_{Hal}, N_{Hdon}	0.43	0.82	0.46	0.73
LUMO, ΔE, logP, N_{Hal}, N_{Hdon}	0.53	0.69	0.66	0.53
LUMO, ΔE, MW, N_{Hal}, N_{Hdon}	0.55	0.69	0.64	0.56
LUMO, ΔE, MW, logP, N_{Hdon}	0.44	0.82	0.47	0.74
LUMO, ΔE, MW, logP, N_{Hal}	0.45	0.82	0.46	0.73

Supplementary Table 4: Molecular descriptors and the obtaining methods

Software	Descriptors
Gaussian 03	HOMO energy, LUMO energy, the HOMO-LUMO gap (ΔE), the total molecular energy (E_{Tot}), the minimum (Q_{Nmax}) and the maximum (Q_{Pmax}) atomic partial charge, dipole moment (μ), polarizability (α)
HyperChem release 7.5	Heat of formation (HF), molecular surface area (MSA), molecular volume (MVol), logarithm of the octanol-water partition coefficient (logP), hydration energy (HE), molecular refractivity (MR)
TSAR V3.3	Molecular weight (MW); Kier and Hall simple and valence-corrected molecular connectivity indices (χ); Kappa shape indices (κ); shape flexibility (Φ); Wiener, Randic and Balaban topological indices; E-state indice (S); the number of H-bond donors (N_{Hdon}) and acceptors (N_{Hacc}); atom counts (oxygen, nitrogen, fluorine, chlorine, bromine, iodine, halogen atoms, heteroatoms); group counts (hydroxyl, amino, aldehyde, nitro, cyano, acid anhydride, methyl)

Supplementary Table 5: Parameters of the GA-SVR feature selection

Parameter	Value	Parameter	Value
Population Size	50	Regression method	SVR
Maximum generations	100	Cross-validation	5-fold
Probability of crossover	0.75	Fitness function	*RMSE*
Probability of mutation	0.01	Regularization parameter (C)	10
