# Supplementary material for: Prediction of the aquatic toxicity of aromatic compounds to tetrahymena pyriformis through support vector regression
Source: Oncotarget. 2017 Apr 13;8(30):49359–69. doi: 10.18632/oncotarget.17210 (PMC5564774; doi:10.18632/oncotarget.17210)
Supplement: Supplementary file 2 [file oncotarget-08-49359-s002.docx]

Supporting Information S1

Table

Chemical name, Chemical Abstract Service (CAS) number, experimental and predicted toxicity values (*logIGC_50_^-1^*) to *Tetrahymena pyriformis*, and calculated descriptors used in SVR model

| **ID** | **Name** | **CAS number** | ***log(IGC_50_^-1^)*** | | **LUMO** | **ΔE** | **MW** | **logP ^b^** | **N_Hal_** | **N_Hdon_** |
| --- | --- | --- | --- | --- | --- | --- | --- | --- | --- | --- |
|  |  |  | **observed** | **Predicted ^a^** |  |  |  |  |  |  |
| *Compounds for QSAR development* | | | | | | | | | | |
| 1 | 3-amino-4-hydroxybenzenesulfonic acid | 98-37-3 | -1.053 | -0.687 | -0.044 | -0.209 | 189.20 | 0.19 ^c^ | 0 | 3 |
| 2 | benzene-1,3,5-triol | 108-73-6 | -1.260 | -0.952 | 0.010 | -0.231 | 126.12 | 0.16 ^m^ | 0 | 3 |
| 3 | 5-(hydroxymethyl)-2-methoxyphenol | 4383-06-6 | -0.990 | -0.250 | 0.000 | -0.208 | 154.18 | 0.97 ^c^ | 0 | 2 |
| 4 | 4-(aminomethyl)-2-methoxyphenol | 7149-10-2 | -0.970 | -0.401 | 0.002 | -0.206 | 153.20 | 0.63 ^c^ | 0 | 2 |
| 5 | 2-(hydroxymethyl)phenol | 90-01-7 | -0.954 | -0.611 | -0.003 | -0.219 | 124.15 | 0.73 ^m^ | 0 | 2 |
| 6 | 3-(2-Hydroxyethoxy)phenol | 49650-88-6 | -0.870 | -0.336 | -0.002 | -0.215 | 154.18 | 1.07 ^c^ | 0 | 2 |
| 7 | 4-(2-hydroxyethyl)phenol | 501-94-0 | -0.826 | -0.064 | -0.012 | -0.211 | 138.18 | 1.48 ^c^ | 0 | 2 |
| 8 | N-(4-hydroxyphenyl)acetamide | 103-90-2 | -0.820 | -0.499 | -0.028 | -0.199 | 151.18 | 0.32 ^m^ | 0 | 2 |
| 9 | 4-(hydroxymethyl)-2-methoxyphenol | 498-00-0 | -0.700 | -0.211 | 0.000 | -0.206 | 154.18 | 0.97 ^c^ | 0 | 2 |
| 10 | benzene-1,3-diol | 108-46-3 | -0.650 | -0.523 | -0.005 | -0.216 | 110.12 | 0.80 ^m^ | 0 | 2 |
| 11 | 2,6-dimethoxyphenol | 91-10-1 | -0.598 | -0.328 | 0.009 | -0.210 | 154.18 | 1.15 ^m^ | 0 | 1 |
| 12 | 3-aminophenol | 591-27-5 | -0.524 | -0.616 | 0.002 | -0.205 | 109.14 | 0.23 ^m^ | 0 | 2 |
| 13 | 2,4,6-tris(dimethylaminomethyl) phenol | 90-72-2 | -0.520 | -0.133 | 0.005 | -0.211 | 265.45 | 1.42 ^c^ | 0 | 1 |
| 14 | 2-methoxyphenol | 90-05-1 | -0.510 | -0.328 | 0.000 | -0.212 | 124.15 | 1.32 ^m^ | 0 | 1 |
| 15 | 4-methyl-3-nitropyridin-2-ol | 21901-18-9 | -0.500 | 0.651 | -0.113 | -0.157 | 154.14 | 2.04 ^c^ | 0 | 1 |
| 16 | 3,6-dihydroxyphthalonitrile | 4733-50-0 | -0.441 | 0.468 | -0.098 | -0.156 | 160.14 | 1.21 ^c^ | 0 | 2 |
| 17 | 5-methylbenzene-1,3-diol | 504-15-4 | -0.390 | 0.176 | -0.002 | -0.216 | 124.15 | 1.95 ^c^ | 0 | 2 |
| 18 | 1-(3-hydroxyphenyl)ethanone | 121-71-1 | -0.381 | 0.113 | -0.062 | -0.177 | 136.16 | 1.39 ^m^ | 0 | 1 |
| 19 | 2-(4-hydroxyphenyl)acetonitrile | 14191-95-8 | -0.384 | -0.087 | -0.049 | -0.202 | 133.16 | 1.56 ^c^ | 0 | 1 |
| 20 | 2-ethoxyphenol | 94-71-3 | -0.358 | -0.067 | 0.000 | -0.211 | 138.18 | 1.68 ^m^ | 0 | 1 |
| 21 | 1-(4-hydroxyphenyl)ethanone | 99-93-4 | -0.302 | -0.018 | -0.054 | -0.188 | 136.16 | 1.35 ^m^ | 0 | 1 |
| 22 | 3-ethoxy-4-methoxyphenol | 65383-57-5 | -0.299 | 0.144 | -0.007 | -0.196 | 168.21 | 1.60 ^c^ | 0 | 1 |
| 23 | 2-methylphenol | 95-48-7 | -0.295 | -0.079 | -0.005 | -0.218 | 108.15 | 1.96 ^m^ | 0 | 1 |
| 24 | 2-hydroxybenzamide | 65-45-2 | -0.242 | 0.196 | -0.052 | -0.179 | 137.15 | 1.28 ^m^ | 0 | 2 |
| 25 | ethyl 2-(4-hydroxy-3-methoxyphenyl)acetate | 60563-13-5 | -0.230 | 0.083 | -0.011 | -0.201 | 210.25 | 1.52 ^c^ | 0 | 1 |
| 26 | phenol | 108-95-2 | -0.208 | -0.422 | -0.011 | -0.217 | 94.12 | 1.46 ^m^ | 0 | 1 |
| 27 | 4-(2-hydroxyethyl)-2-methoxyphenol | 2380-78-1 | -0.180 | -0.614 | -0.002 | -0.211 | 168.21 | 0.47 ^m^ | 0 | 2 |
| 28 | 2-hydroxy-4-methyl-5-nitropyridine | 21901-41-7 | -0.170 | 0.487 | -0.094 | -0.178 | 154.14 | 2.04 ^c^ | 0 | 1 |
| 29 | N-(3-hydroxyphenyl)acetamide | 621-42-1 | -0.155 | -0.398 | -0.027 | -0.205 | 151.18 | 0.73 ^m^ | 0 | 2 |
| 30 | 4-methoxyphenol | 150-76-5 | -0.143 | -0.077 | -0.012 | -0.193 | 124.15 | 1.34 ^m^ | 0 | 1 |
| 31 | 1-(4-hydroxy-3-methoxyphenyl)ethanone | 498-02-2 | -0.120 | 0.040 | -0.052 | -0.173 | 166.19 | 0.82 ^c^ | 0 | 1 |
| 32 | 3,5-dimethoxyphenol | 500-99-2 | -0.092 | -0.305 | 0.012 | -0.228 | 154.18 | 1.64 ^m^ | 0 | 1 |
| 33 | 2-hydroxyethyl 2-hydroxybenzoate | 87-28-5 | -0.080 | 0.155 | -0.049 | -0.181 | 182.19 | 1.05 ^c^ | 0 | 2 |
| 34 | 3-methylphenol | 108-39-4 | -0.063 | -0.074 | -0.007 | -0.217 | 108.15 | 1.96 ^m^ | 0 | 1 |
| 35 | 3-hydroxybenzonitrile | 873-62-1 | -0.064 | 0.003 | -0.060 | -0.194 | 119.13 | 1.70 ^m^ | 0 | 1 |
| 36 | methyl-3-hydroxybenzoate | 19438-10-9 | -0.046 | 0.313 | -0.053 | -0.185 | 152.16 | 1.89 ^m^ | 0 | 1 |
| 37 | 4-ethoxyphenol | 622-62-8 | 0.013 | 0.226 | -0.011 | -0.193 | 138.18 | 1.81 ^m^ | 0 | 1 |
| 38 | 4-fluorophenol | 371-41-5 | 0.017 | 0.142 | -0.024 | -0.205 | 112.11 | 1.77 ^m^ | 1 | 1 |
| 39 | 1-(5-fluoro-2-hydroxyphenyl)ethanone | 394-32-1 | 0.040 | 0.296 | -0.067 | -0.176 | 154.15 | 1.21 ^c^ | 1 | 1 |
| 40 | 1-(4-hydroxyphenyl)propan-1-one | 70-70-2 | 0.053 | 0.337 | -0.053 | -0.188 | 150.19 | 2.03 ^m^ | 0 | 1 |
| 41 | 2,4-dimethylphenol | 105-67-9 | 0.070 | 0.267 | -0.005 | -0.210 | 122.18 | 2.30 ^m^ | 0 | 1 |
| 42 | 2,5-dimethylphenol | 95-87-4 | 0.081 | 0.237 | -0.005 | -0.214 | 122.18 | 2.34 ^m^ | 0 | 1 |
| 43 | methyl-4-hydroxybenzoate | 99-76-3 | 0.084 | 0.212 | -0.042 | -0.198 | 152.16 | 1.96 ^m^ | 0 | 1 |
| 44 | 3,5-dimethylphenol | 108-68-9 | 0.113 | 0.216 | -0.005 | -0.216 | 122.18 | 2.35 ^m^ | 0 | 1 |
| 45 | 2,3-dimethylphenol | 526-75-0 | 0.122 | 0.308 | -0.005 | -0.215 | 122.18 | 2.48 ^m^ | 0 | 1 |
| 46 | 2,4-diaminophenol | 95-86-3 | 0.127 | 0.196 | -0.008 | -0.179 | 124.16 | 0.20 ^c^ | 0 | 3 |
| 47 | 4-chlorobenzene-1,3-diol | 95-88-5 | 0.130 | 0.459 | -0.020 | -0.208 | 144.56 | 1.80 ^m^ | 1 | 2 |
| 48 | 2-ethylphenol | 90-00-6 | 0.160 | 0.266 | -0.004 | -0.218 | 122.18 | 2.47 ^m^ | 0 | 1 |
| 49 | 4-hydroxy-3,5-dimethoxybenzaldehyde | 134-96-3 | 0.168 | 0.294 | -0.058 | -0.161 | 182.19 | 0.93 ^c^ | 0 | 1 |
| 50 | 2-hydroxybenzohydrazide | 936-02-7 | 0.181 | 0.188 | -0.045 | -0.176 | 152.17 | 0.60 ^m^ | 0 | 3 |
| 51 | 2-fluorophenol | 367-12-4 | 0.185 | -0.007 | -0.015 | -0.218 | 112.11 | 1.71 ^m^ | 1 | 1 |
| 52 | 1-(4-hydroxy-2-methylphenyl)ethanone | 875-59-2 | 0.190 | 0.131 | -0.052 | -0.187 | 150.19 | 1.54 ^c^ | 0 | 1 |
| 53 | 4-ethylphenol | 123-07-9 | 0.205 | 0.347 | -0.009 | -0.210 | 122.18 | 2.47 ^m^ | 0 | 1 |
| 54 | 3-ethylphenol | 620-17-7 | 0.228 | 0.241 | -0.009 | -0.215 | 122.18 | 2.40 ^m^ | 0 | 1 |
| 55 | salicylaldoxime | 94-67-7 | 0.253 | 0.586 | -0.051 | -0.176 | 137.15 | 2.10 ^c^ | 0 | 2 |
| 56 | 3,4-dinitrophenol | 577-71-9 | 0.266 | 0.822 | -0.111 | -0.169 | 184.12 | 2.23 ^m^ | 0 | 1 |
| 57 | 2,4,6-trimethylphenol | 527-60-6 | 0.281 | 0.570 | 0.000 | -0.211 | 136.21 | 2.73 ^m^ | 0 | 1 |
| 58 | 3-methylbenzene-1,2-diol | 488-17-5 | 0.280 | 0.256 | -0.001 | -0.212 | 124.15 | 1.95 ^c^ | 0 | 2 |
| 59 | 1-(2-hydroxy-5-methylphenyl)ethanone | 1450-72-2 | 0.310 | 0.215 | -0.055 | -0.179 | 150.19 | 1.54 ^c^ | 0 | 1 |
| 60 | 2-prop-2-enylphenol | 1745-81-9 | 0.334 | 0.489 | -0.008 | -0.217 | 134.19 | 2.81 ^c^ | 0 | 1 |
| 61 | 2-bromophenol | 95-56-7 | 0.330 | 0.455 | -0.025 | -0.213 | 173.01 | 2.35 ^m^ | 1 | 1 |
| 62 | 4-bromo-2-(hydroxymethyl)phenol | 2316-64-5 | 0.343 | 0.378 | -0.018 | -0.207 | 203.04 | 1.72 ^m^ | 1 | 2 |
| 63 | 2-amino-4-tert-butylphenol | 1199-46-8 | 0.366 | 0.945 | 0.002 | -0.196 | 165.26 | 2.61 ^c^ | 0 | 2 |
| 64 | 4-methylbenzene-1,2-diol | 452-86-8 | 0.370 | 0.004 | -0.004 | -0.206 | 124.15 | 1.37 ^m^ | 0 | 2 |
| 65 | salicylhydroxamic acid | 89-73-6 | 0.379 | 0.392 | -0.061 | -0.171 | 153.15 | 1.08 ^c^ | 0 | 3 |
| 66 | 3-fluorophenol | 372-20-3 | 0.381 | 0.077 | -0.016 | -0.221 | 112.11 | 1.93 ^m^ | 1 | 1 |
| 67 | 2-chloro-5-methylphenol | 615-74-7 | 0.393 | 0.656 | -0.023 | -0.209 | 142.59 | 2.75 ^c^ | 1 | 1 |
| 68 | 6-methyl-2-nitropyridin-3-ol | 15128-90-2 | 0.390 | 0.354 | -0.080 | -0.178 | 154.14 | 1.75 ^c^ | 0 | 1 |
| 69 | benzene-1,2,4-triol | 533-73-3 | 0.440 | 0.367 | -0.005 | -0.196 | 126.12 | 1.19 ^c^ | 0 | 3 |
| 70 | 5-amino-2-methoxyphenol | 1687-53-2 | 0.450 | -0.029 | 0.004 | -0.191 | 139.17 | 0.73 ^c^ | 0 | 2 |
| 71 | 2,3-dinitrophenol | 66-56-8 | 0.463 | 0.715 | -0.112 | -0.163 | 184.12 | 1.67 ^c^ | 0 | 1 |
| 72 | 2,6-difluorophenol | 28177-48-2 | 0.471 | 0.394 | -0.016 | -0.226 | 130.10 | 2.05 ^m^ | 2 | 1 |
| 73 | 4-propan-2-ylphenol | 99-89-8 | 0.473 | 0.626 | -0.010 | -0.210 | 136.21 | 2.90 ^m^ | 0 | 1 |
| 74 | benzene-1,4-diol | 123-31-9 | 0.470 | -0.060 | -0.014 | -0.194 | 110.12 | 0.99 ^m^ | 0 | 2 |
| 75 | 2-amino-4-nitrophenol | 99-57-0 | 0.475 | 0.423 | -0.086 | -0.167 | 154.14 | 1.53 ^m^ | 0 | 2 |
| 76 | 3-nitrophenol | 554-84-7 | 0.506 | 0.484 | -0.095 | -0.162 | 139.12 | 2.00 ^m^ | 0 | 1 |
| 77 | 4-hydroxybenzonitrile | 767-00-0 | 0.516 | -0.124 | -0.049 | -0.202 | 119.13 | 1.60 ^m^ | 0 | 1 |
| 78 | 4-propyloxyphenol | 18979-50-5 | 0.520 | 0.550 | -0.011 | -0.193 | 152.21 | 2.33 ^m^ | 0 | 1 |
| 79 | 2,6-dinitrophenol | 573-56-8 | 0.539 | 0.797 | -0.129 | -0.154 | 184.12 | 1.37 ^m^ | 0 | 1 |
| 80 | 1-(2-hydroxy-4-methoxyphenyl)ethanone | 552-41-0 | 0.550 | 0.399 | -0.045 | -0.186 | 166.19 | 1.98 ^m^ | 0 | 1 |
| 81 | ethyl-4-hydroxybenzoate | 120-47-8 | 0.573 | 0.504 | -0.041 | -0.198 | 166.19 | 2.47 ^m^ | 0 | 1 |
| 82 | 4-methyl-2-nitrophenol | 119-33-5 | 0.571 | 0.653 | -0.083 | -0.166 | 153.15 | 2.37 ^m^ | 0 | 1 |
| 83 | 5-methyl-2-nitrophenol | 700-38-9 | 0.590 | 0.718 | -0.102 | -0.152 | 153.15 | 2.31 ^m^ | 0 | 1 |
| 84 | 2-bromo-4-methylphenol | 6627-55-0 | 0.599 | 0.875 | -0.023 | -0.206 | 187.04 | 3.02 ^c^ | 1 | 1 |
| 85 | 2,4-difluorophenol | 367-27-1 | 0.604 | 0.264 | -0.028 | -0.208 | 130.10 | 1.46 ^m^ | 2 | 1 |
| 86 | 3-propan-2-ylphenol | 618-45-1 | 0.612 | 0.588 | -0.007 | -0.217 | 136.21 | 2.96 ^c^ | 0 | 1 |
| 87 | 4-(trifluoromethyl)phenol | 402-45-9 | 0.618 | 1.070 | -0.031 | -0.219 | 162.12 | 2.82 ^m^ | 3 | 1 |
| 88 | methyl 2-hydroxy-4-methoxybenzoate | 5446-02-6 | 0.620 | 0.090 | -0.044 | -0.188 | 182.19 | 1.24 ^c^ | 0 | 1 |
| 89 | 2,6-dichloro-4-nitrophenol | 618-80-4 | 0.632 | 1.235 | -0.106 | -0.169 | 208.00 | 2.94 ^m^ | 2 | 1 |
| 90 | 4-propylphenol | 645-56-7 | 0.643 | 0.684 | -0.010 | -0.210 | 136.21 | 3.00 ^m^ | 0 | 1 |
| 91 | 4-nitrosophenol | 104-91-6 | 0.654 | 0.420 | -0.097 | -0.121 | 123.12 | 1.29 ^m^ | 0 | 1 |
| 92 | 2-nitrobenzene-1,3-diol | 601-89-8 | 0.660 | 0.769 | -0.116 | -0.129 | 155.12 | 1.56 ^m^ | 0 | 2 |
| 93 | 2-nitrophenol | 88-75-5 | 0.670 | 0.495 | -0.106 | -0.152 | 139.12 | 1.79 ^m^ | 0 | 1 |
| 94 | 4-bromophenol | 106-41-2 | 0.680 | 0.659 | -0.027 | -0.204 | 173.01 | 2.59 ^m^ | 1 | 1 |
| 95 | 2-chloro-4,5-dimethylphenol | 1124-04-5 | 0.688 | 0.940 | -0.017 | -0.209 | 156.62 | 3.22 ^c^ | 1 | 1 |
| 96 | 4-chloro-2-methylphenol | 1570-64-5 | 0.701 | 0.703 | -0.022 | -0.206 | 142.59 | 2.78 ^m^ | 1 | 1 |
| 97 | 4-butoxyphenol | 122-94-1 | 0.701 | 0.912 | -0.011 | -0.192 | 166.24 | 2.90 ^m^ | 0 | 1 |
| 98 | 1-(2-hydroxy-4,5-dimethylphenyl)ethanone | 36436-65-4 | 0.707 | 0.463 | -0.051 | -0.180 | 164.22 | 2.01 ^c^ | 0 | 1 |
| 99 | 4-methyl-3-nitrophenol | 2042-14-0 | 0.740 | 0.643 | -0.090 | -0.158 | 153.15 | 2.18 ^c^ | 0 | 1 |
| 100 | 2,6-dichlorophenol | 87-65-0 | 0.735 | 0.794 | -0.037 | -0.210 | 163.00 | 2.64 ^m^ | 2 | 1 |
| 101 | 2-(chloromethyl)-4-nitrophenol | 2973-19-5 | 0.750 | 0.844 | -0.091 | -0.176 | 187.59 | 2.33 ^c^ | 1 | 1 |
| 102 | 2-methoxy-4-[(E)-prop-1-enyl]phenol | 97-54-1 | 0.750 | 1.129 | -0.024 | -0.173 | 164.22 | 3.04 ^m^ | 0 | 1 |
| 103 | benzene-1,2-diol | 120-80-9 | 0.750 | -0.356 | -0.004 | -0.212 | 110.12 | 0.95 ^m^ | 0 | 2 |
| 104 | 3-chloro-5-methoxyphenol | 65262-96-6 | 0.757 | 0.237 | -0.018 | -0.217 | 158.59 | 2.03 ^c^ | 1 | 1 |
| 105 | 2-methyl-3-nitrophenol | 5460-31-1 | 0.780 | 0.617 | -0.087 | -0.162 | 153.15 | 2.18 ^c^ | 0 | 1 |
| 106 | 4-chloro-3-methylphenol | 59-50-7 | 0.796 | 0.860 | -0.021 | -0.207 | 142.59 | 3.10 ^m^ | 1 | 1 |
| 107 | 2-propan-2-ylphenol | 88-69-7 | 0.798 | 0.553 | -0.007 | -0.216 | 136.21 | 2.88 ^m^ | 0 | 1 |
| 108 | 2,6-dichloro-4-fluorophenol | 392-71-2 | 0.804 | 1.180 | -0.048 | -0.200 | 180.99 | 2.94 ^c^ | 3 | 1 |
| 109 | 2,5-dichlorobenzene-1,4-diol | 824-69-1 | 1.810 | 1.232 | -0.039 | -0.188 | 179.00 | 2.51 ^c^ | 2 | 2 |
| 110 | 4-iodophenol | 540-38-5 | 0.854 | 0.964 | -0.035 | -0.192 | 220.01 | 2.91 ^m^ | 1 | 1 |
| 111 | 3-chlorophenol | 108-43-0 | 0.871 | 0.444 | -0.025 | -0.215 | 128.56 | 2.50 ^m^ | 1 | 1 |
| 112 | 2-bromo-N-(2-hydroxy-5-nitrophenyl)acetamide | 3947-58-8 | 0.870 | 1.002 | -0.102 | -0.154 | 275.07 | 1.22 ^c^ | 1 | 2 |
| 113 | 2-nitropyridin-3-ol | 15128-82-2 | 0.870 | 0.407 | -0.110 | -0.162 | 140.11 | 1.57 ^c^ | 0 | 1 |
| 114 | 4-amino-2-nitrophenol | 119-34-6 | 0.879 | 0.768 | -0.100 | -0.118 | 154.14 | 0.96 ^m^ | 0 | 2 |
| 115 | 2,2'-biphenol | 1806-29-7 | 0.880 | 1.065 | -0.020 | -0.195 | 186.22 | 3.16 ^c^ | 0 | 2 |
| 116 | phenyl-(2,3,4-trihydroxyphenyl)methanone | 1143-72-2 | 0.880 | 0.933 | -0.059 | -0.167 | 230.23 | 2.42 ^c^ | 0 | 3 |
| 117 | 4-tert-butylphenol | 98-54-4 | 0.914 | 0.888 | -0.009 | -0.211 | 150.24 | 3.31 ^m^ | 0 | 1 |
| 118 | 3,4,5-trimethylphenol | 527-54-8 | 0.932 | 0.821 | 0.000 | -0.212 | 136.21 | 3.16 ^c^ | 0 | 1 |
| 119 | 3-fluoro-4-nitrophenol | 394-41-2 | 0.940 | 0.571 | -0.091 | -0.179 | 157.11 | 1.86 ^c^ | 1 | 1 |
| 120 | 2,5-dinitrophenol | 329-71-5 | 0.950 | 0.965 | -0.139 | -0.143 | 184.12 | 1.75 ^m^ | 0 | 1 |
| 121 | bis(2,4-dihydroxyphenyl)methanone | 131-55-5 | 0.960 | 0.707 | -0.056 | -0.172 | 246.23 | 2.13 ^c^ | 0 | 4 |
| 122 | 4,6-dichlorobenzene-1,3-diol | 137-19-9 | 0.970 | 1.107 | -0.034 | -0.202 | 179.00 | 2.51 ^c^ | 2 | 2 |
| 123 | 4-sec-butylphenol | 99-71-8 | 0.979 | 0.900 | -0.010 | -0.210 | 150.24 | 3.32 ^m^ | 0 | 1 |
| 124 | 3,4,5,6-tetrabromobenzene-1,2-diol | 488-47-1 | 0.980 | 1.400 | -0.074 | -0.166 | 425.68 | 4.65 ^c^ | 4 | 2 |
| 125 | 3-(phenylamino)phenol | 101-18-8 | 1.010 | 1.196 | -0.016 | -0.180 | 185.24 | 2.88 ^c^ | 0 | 2 |
| 126 | (4-hydroxyphenyl)-phenylmethanone | 1137-42-4 | 1.020 | 1.004 | -0.065 | -0.174 | 198.23 | 2.91 ^m^ | 0 | 1 |
| 127 | 2,4-dichlorophenol | 120-83-2 | 1.036 | 1.011 | -0.040 | -0.202 | 163.00 | 3.06 ^m^ | 2 | 1 |
| 128 | 4-(phenylmethoxy)phenol | 103-16-2 | 1.040 | 0.979 | -0.019 | -0.205 | 200.25 | 3.29 ^c^ | 0 | 1 |
| 129 | 4-chlorobenzene-1,2-diol | 2138-22-9 | 1.060 | 0.619 | -0.019 | -0.204 | 144.56 | 2.00 ^c^ | 1 | 2 |
| 130 | 1-(4-hydroxyphenyl)-2-phenylethanone | 2491-32-9 | 1.070 | 0.953 | -0.055 | -0.186 | 212.26 | 2.92 ^c^ | 0 | 1 |
| 131 | 2-fluoro-4-nitrophenol | 403-19-0 | 1.070 | 0.625 | -0.097 | -0.171 | 157.11 | 1.86 ^c^ | 1 | 1 |
| 132 | 2,4-dinitrophenol | 51-28-5 | 1.077 | 0.642 | -0.109 | -0.179 | 184.12 | 1.79 ^m^ | 0 | 1 |
| 133 | 4-chloro-3-ethylphenol | 14143-32-9 | 1.081 | 0.899 | -0.019 | -0.208 | 156.62 | 3.14 ^c^ | 1 | 1 |
| 134 | 2-phenylphenol | 90-43-7 | 1.090 | 0.932 | -0.030 | -0.191 | 170.22 | 3.09 ^m^ | 0 | 1 |
| 135 | 3-iodophenol | 626-02-8 | 1.119 | 0.889 | -0.036 | -0.201 | 220.01 | 2.93 ^m^ | 1 | 1 |
| 136 | 5-fluoro-2-nitrophenol | 446-36-6 | 1.125 | 0.723 | -0.110 | -0.158 | 157.11 | 1.91 ^m^ | 1 | 1 |
| 137 | 3-chloro-4-fluorophenol | 2613-23-2 | 1.131 | 0.729 | -0.036 | -0.203 | 146.55 | 2.42 ^c^ | 2 | 1 |
| 138 | 2,5-dichlorophenol | 583-78-8 | 1.125 | 0.989 | -0.037 | -0.207 | 163.00 | 3.06 ^m^ | 2 | 1 |
| 139 | 3-bromophenol | 591-20-8 | 1.145 | 0.593 | -0.026 | -0.213 | 173.01 | 2.63 ^m^ | 1 | 1 |
| 140 | 6-tert-butyl-2,4-dimethylphenol | 1879-09-0 | 1.157 | 1.584 | -0.002 | -0.211 | 178.30 | 4.32 ^c^ | 0 | 1 |
| 141 | 2-amino-4-chloro-5-nitrophenol | 6358-07-2 | 1.174 | 0.830 | -0.087 | -0.158 | 188.58 | 1.45 ^c^ | 1 | 2 |
| 142 | 4-bromo-2,6-dimethylphenol | 2374-05-2 | 1.167 | 1.139 | -0.015 | -0.207 | 201.07 | 3.49 ^c^ | 1 | 1 |
| 143 | 4-nitrobenzene-1,2-diol | 3316-09-4 | 1.170 | 0.549 | -0.089 | -0.159 | 155.12 | 1.66 ^m^ | 0 | 2 |
| 144 | 4,6-dinitro-1,2,3-trihydroxybenzene | 3264-71-9 | 1.180 | 0.750 | -0.121 | -0.145 | 216.12 | 1.10 ^c^ | 0 | 3 |
| 145 | 4-chloro-3,5-dimethylphenol | 88-04-0 | 1.201 | 0.966 | -0.015 | -0.210 | 156.62 | 3.27 ^m^ | 1 | 1 |
| 146 | 4-(2-methylbutan-2-yl)phenol | 80-46-6 | 1.229 | 1.222 | -0.010 | -0.210 | 164.27 | 3.83 ^m^ | 0 | 1 |
| 147 | (2-hydroxyphenyl)-phenylmethanone | 117-99-7 | 1.230 | 1.308 | -0.076 | -0.161 | 198.23 | 3.52 ^m^ | 0 | 1 |
| 148 | 2-tert-butylphenol | 88-18-6 | 1.295 | 0.811 | -0.007 | -0.218 | 150.24 | 3.31 ^m^ | 0 | 1 |
| 149 | 2-chlorobenzene-1,4-diol | 615-67-8 | 1.260 | 0.480 | -0.028 | -0.191 | 144.56 | 1.40 ^m^ | 1 | 2 |
| 150 | 4-chloro-3-nitrophenol | 610-78-6 | 1.270 | 0.845 | -0.098 | -0.158 | 173.56 | 2.23 ^c^ | 1 | 1 |
| 151 | 4-bromo-3,5-dimethylphenol | 7463-51-6 | 1.268 | 1.118 | -0.015 | -0.209 | 201.07 | 3.49 ^c^ | 1 | 1 |
| 152 | 4-bromo-6-chloro-2-methylphenol | 7530-27-0 | 1.276 | 1.232 | -0.032 | -0.204 | 221.48 | 3.54 ^c^ | 2 | 1 |
| 153 | 2,3,5,6-tetramethylbenzene-1,4-diol | 527-18-4 | 1.280 | 0.469 | -0.001 | -0.195 | 166.24 | 1.80 ^m^ | 0 | 2 |
| 154 | 4-cyclopentylphenol | 1518-83-8 | 1.292 | 0.905 | -0.010 | -0.208 | 162.25 | 3.25 ^c^ | 0 | 1 |
| 155 | 2-tert-butyl-4-methylphenol | 2409-55-4 | 1.301 | 1.262 | -0.007 | -0.210 | 164.27 | 3.86 ^c^ | 0 | 1 |
| 156 | 5-pentylbenzene-1,3-diol | 500-66-3 | 1.310 | 1.084 | -0.002 | -0.216 | 180.27 | 3.53 ^c^ | 0 | 2 |
| 157 | 2-(phenylmethyl)phenol | 28994-41-4 | 1.310 | 1.266 | -0.018 | -0.205 | 184.25 | 3.84 ^c^ | 0 | 1 |
| 158 | 4-amino-2-methyl-phenol | 2635-95-2 | 1.307 | 0.441 | -0.003 | -0.185 | 123.17 | 1.45 ^c^ | 0 | 2 |
| 159 | butyl-4-hydroxybenzoate | 94-26-8 | 1.330 | 1.112 | -0.041 | -0.198 | 194.25 | 3.57 ^m^ | 0 | 1 |
| 160 | 2,3,5-trimethylbenzene-1,4-diol | 700-13-0 | 1.340 | 0.427 | -0.003 | -0.194 | 152.21 | 1.71 ^m^ | 0 | 2 |
| 161 | 2,6-dibromo-4-nitrophenol | 99-28-5 | 1.356 | 1.561 | -0.104 | -0.166 | 296.90 | 3.57 ^m^ | 2 | 1 |
| 162 | 3-phenylphenol | 580-51-8 | 1.350 | 0.990 | -0.035 | -0.190 | 170.22 | 3.23 ^m^ | 0 | 1 |
| 163 | 4-pentyloxyphenol | 18979-53-8 | 1.360 | 1.079 | -0.014 | -0.206 | 180.27 | 3.50 ^m^ | 0 | 1 |
| 164 | 2-nitronaphthalen-1-ol | 607-24-9 | 1.360 | 1.131 | -0.103 | -0.134 | 189.18 | 2.72 ^c^ | 0 | 1 |
| 165 | 4-fluoro-2-nitrophenol | 394-33-2 | 1.380 | 0.765 | -0.115 | -0.143 | 157.11 | 1.88 ^m^ | 1 | 1 |
| 166 | 4-phenylphenol | 92-69-3 | 1.390 | 1.032 | -0.028 | -0.188 | 170.22 | 3.20 ^m^ | 0 | 1 |
| 167 | 3,5-dinitrophenol | 586-11-8 | 1.390 | 0.931 | -0.122 | -0.161 | 184.12 | 2.34 ^m^ | 0 | 1 |
| 168 | 2,4-dibromophenol | 615-58-7 | 1.398 | 1.112 | -0.039 | -0.200 | 251.90 | 3.22 ^m^ | 2 | 1 |
| 169 | 2,4,6-trichlorophenol | 88-06-2 | 1.410 | 1.431 | -0.050 | -0.200 | 197.44 | 3.69 ^m^ | 3 | 1 |
| 170 | 2,3-dimethylbenzene-1,4-diol | 608-43-5 | 1.410 | 0.203 | -0.007 | -0.194 | 138.18 | 1.36 ^m^ | 0 | 2 |
| 171 | (2-hydroxy-4-methoxyphenyl)-phenylmethanone | 131-57-7 | 1.420 | 1.544 | -0.069 | -0.162 | 228.26 | 3.82 ^m^ | 0 | 1 |
| 172 | 4-amino-2,3-dimethylphenol | 3096-69-3 | 1.440 | 0.685 | -0.001 | -0.187 | 137.20 | 1.91 ^c^ | 0 | 2 |
| 173 | isoamyl-4-hydroxybenzoate | 6521-30-8 | 1.480 | 0.883 | -0.041 | -0.198 | 208.28 | 3.03 ^c^ | 0 | 1 |
| 174 | 3,5-dichloro-2-hydroxybenzaldehyde | 90-60-8 | 1.550 | 1.028 | -0.089 | -0.168 | 191.01 | 2.47 ^c^ | 2 | 1 |
| 175 | benzyl-4-hydroxybenzoate | 94-18-8 | 1.550 | 1.187 | -0.042 | -0.197 | 228.26 | 3.56 ^m^ | 0 | 1 |
| 176 | 3,5-dichlorophenol | 591-35-5 | 1.569 | 1.186 | -0.038 | -0.214 | 163.00 | 3.62 ^m^ | 2 | 1 |
| 177 | 2-chloro-4-nitrophenol | 619-08-9 | 1.585 | 0.800 | -0.098 | -0.171 | 173.56 | 2.23 ^c^ | 1 | 1 |
| 178 | 4-bromo-2-fluoro-6-nitrophenol | 320-76-3 | 1.620 | 1.350 | -0.122 | -0.138 | 236.00 | 2.65 ^c^ | 2 | 1 |
| 179 | 2,3,4,5,6-pentafluorophenol | 771-61-9 | 1.638 | 1.801 | -0.033 | -0.226 | 184.07 | 3.23 ^m^ | 5 | 1 |
| 180 | 4-chloro-6-nitro-3-methylphenol | 7147-89-9 | 1.638 | 1.120 | -0.111 | -0.144 | 187.59 | 2.93 ^m^ | 1 | 1 |
| 181 | 3,5-ditert-butylphenol | 1138-52-9 | 1.638 | 1.548 | -0.005 | -0.215 | 206.36 | 4.35 ^m^ | 0 | 1 |
| 182 | 4-hexyloxyphenol | 18979-55-0 | 1.638 | 1.104 | -0.014 | -0.206 | 194.30 | 3.51 ^c^ | 0 | 1 |
| 183 | 4-Phenylazophenol | 1689-82-3 | 1.660 | 1.325 | -0.083 | -0.138 | 198.24 | 3.18 ^m^ | 0 | 1 |
| 184 | 2-bromobenzene-1,4-diol | 583-69-7 | 1.680 | 0.856 | -0.028 | -0.191 | 189.01 | 2.27 ^c^ | 1 | 2 |
| 185 | 3,4,5,6-tetrachlorobenzene-1,2-diol | 1198-55-6 | 1.700 | 2.117 | -0.050 | -0.195 | 247.88 | 4.29 ^m^ | 4 | 2 |
| 186 | 2,6-diiodo-4-nitrophenol | 305-85-1 | 1.712 | 1.755 | -0.102 | -0.159 | 390.90 | 4.23 ^c^ | 2 | 1 |
| 187 | 2-methyl-4,6-dinitrophenol | 534-52-1 | 1.721 | 1.022 | -0.106 | -0.175 | 198.15 | 2.86 ^m^ | 0 | 1 |
| 188 | 3-methyl-4-nitrophenol | 2581-34-2 | 1.729 | 0.637 | -0.084 | -0.174 | 153.15 | 2.48 ^m^ | 0 | 1 |
| 189 | 3,4-dichlorophenol | 95-77-2 | 1.745 | 1.118 | -0.037 | -0.204 | 163.00 | 3.33 ^m^ | 2 | 1 |
| 190 | 4-bromo-2,6-dichlorophenol | 3217-15-0 | 1.778 | 1.376 | -0.049 | -0.199 | 241.89 | 3.59 ^c^ | 3 | 1 |
| 191 | 4-hexylbenzene-1,3-diol | 136-77-6 | 1.800 | 1.105 | 0.000 | -0.212 | 194.30 | 3.45 ^m^ | 0 | 2 |
| 192 | 4-tert-butyl-2,6-dinitrophenol | 4097-49-8 | 1.800 | 1.522 | -0.124 | -0.147 | 240.24 | 3.30 ^c^ | 0 | 1 |
| 193 | 2,6-ditert-butyl-4-methylphenol | 128-37-0 | 1.796 | 1.530 | -0.002 | -0.210 | 220.39 | 4.17 ^m^ | 0 | 1 |
| 194 | 2,3,5,6-tetrafluorobenzene-1,4-diol | 771-63-1 | 1.840 | 1.630 | -0.024 | -0.215 | 182.08 | 2.04 ^c^ | 4 | 2 |
| 195 | 4-bromo-2-nitrophenol | 7693-52-9 | 1.870 | 1.214 | -0.116 | -0.140 | 218.01 | 2.51 ^c^ | 1 | 1 |
| 196 | 2,4-dinitronaphth-1-ol | 605-69-6 | 1.890 | 1.390 | -0.122 | -0.138 | 234.18 | 2.67 ^c^ | 0 | 1 |
| 197 | 2-phenylbenzene-1,4-diol | 1079-21-6 | 2.000 | 1.272 | -0.032 | -0.175 | 186.22 | 3.16 ^c^ | 0 | 2 |
| 198 | 2,4,6-tribromophenol | 118-79-6 | 2.030 | 1.456 | -0.057 | -0.189 | 330.79 | 4.13 ^m^ | 3 | 1 |
| 199 | 4-heptyloxyphenol | 13037-86-0 | 2.033 | 1.541 | -0.011 | -0.192 | 208.33 | 3.91 ^c^ | 0 | 1 |
| 200 | 2,3,4,5,6-pentachlorophenol | 87-86-5 | 2.049 | 2.011 | -0.063 | -0.196 | 266.32 | 5.12 ^m^ | 5 | 1 |
| 201 | 2,4,5-trichlorophenol | 95-95-4 | 2.097 | 1.441 | -0.049 | -0.200 | 197.44 | 3.72 ^m^ | 3 | 1 |
| 202 | 4-(2,4,4-trimethylpentan-2-yl)phenol | 140-66-9 | 2.097 | 1.924 | -0.009 | -0.211 | 206.36 | 4.95 ^c^ | 0 | 1 |
| 203 | 3,5-ditert-butylbenzene-1,2-diol | 1020-31-1 | 2.110 | 1.890 | 0.000 | -0.208 | 222.36 | 4.73 ^c^ | 0 | 2 |
| 204 | 2,3,5,6-tetrachlorobenzene-1,4-diol | 87-87-6 | 2.110 | 1.948 | -0.055 | -0.186 | 247.88 | 3.55 ^c^ | 4 | 2 |
| 205 | 2,3,4,6-tetrachlorophenol | 58-90-2 | 2.180 | 1.791 | -0.056 | -0.199 | 231.88 | 4.45 ^m^ | 4 | 1 |
| 206 | 2,3,5,6-tetrachlorophenol | 935-95-5 | 2.222 | 1.639 | -0.055 | -0.205 | 231.88 | 3.88 ^m^ | 4 | 1 |
| 207 | 2-hydroxy-3,5-diiodobenzaldehyde | 2631-77-8 | 2.340 | 1.614 | -0.086 | -0.161 | 373.91 | 3.95 ^c^ | 2 | 1 |
| 208 | 2,3,5-trichlorophenol | 933-78-8 | 2.373 | 1.471 | -0.046 | -0.207 | 197.44 | 3.84 ^m^ | 3 | 1 |
| 209 | 4-nonylphenol | 104-40-5 | 2.468 | 2.433 | -0.009 | -0.209 | 220.39 | 5.76 ^m^ | 0 | 1 |
| 210 | 2-ethylhexyl 4-hydroxybenzoate | 5153-25-3 | 2.510 | 1.566 | -0.041 | -0.198 | 250.37 | 4.29 ^c^ | 0 | 1 |
| 211 | 3,4,5,6-tetrabromo-2-methylphenol | 576-55-6 | 2.574 | 1.472 | -0.067 | -0.176 | 423.71 | 5.40 ^c^ | 4 | 1 |
| 212 | nonyl-4-hydroxybenzoate | 38713-56-3 | 2.630 | 1.771 | -0.041 | -0.198 | 264.40 | 4.68 ^c^ | 0 | 1 |
| 213 | 2,3,4,5-tetrachlorophenol | 4901-51-3 | 2.712 | 1.954 | -0.056 | -0.199 | 231.88 | 5.05 ^m^ | 4 | 1 |
| 214 | (3-aminophenyl)methanol | 1877-77-6 | -1.130 | -0.690 | -0.003 | -0.200 | 123.17 | -0.05 ^m^ | 0 | 2 |
| 215 | 2,6-dimethylaniline | 87-62-7 | -0.430 | 0.109 | 0.005 | -0.205 | 121.20 | 1.84 ^m^ | 0 | 1 |
| 216 | 2-fluoroaniline | 348-54-9 | -0.370 | -0.076 | -0.008 | -0.204 | 111.13 | 1.26 ^m^ | 1 | 1 |
| 217 | 3-methylaniline | 108-44-1 | -0.280 | -0.185 | 0.000 | -0.203 | 107.17 | 1.40 ^m^ | 0 | 1 |
| 218 | aniline | 62-53-3 | -0.230 | -0.533 | -0.003 | -0.203 | 93.14 | 0.90 ^m^ | 0 | 1 |
| 219 | 2-chloroaniline | 95-51-2 | -0.170 | 0.312 | -0.016 | -0.200 | 127.58 | 1.90 ^m^ | 1 | 1 |
| 220 | 2-methylaniline | 95-53-4 | -0.160 | -0.192 | 0.001 | -0.205 | 107.17 | 1.43 ^m^ | 0 | 1 |
| 221 | 3,4-dimethylaniline | 95-64-7 | -0.160 | 0.194 | 0.002 | -0.199 | 121.20 | 1.86 ^m^ | 0 | 1 |
| 222 | 4-methylaniline | 106-49-0 | -0.050 | -0.101 | -0.002 | -0.197 | 107.17 | 1.41 ^m^ | 0 | 1 |
| 223 | 3-ethylaniline | 587-02-0 | -0.030 | 0.296 | -0.001 | -0.202 | 121.20 | 2.13 ^c^ | 0 | 1 |
| 224 | 4-ethylaniline | 589-16-2 | 0.030 | 0.254 | -0.003 | -0.197 | 121.20 | 1.96 ^m^ | 0 | 1 |
| 225 | benzene-1,4-diamine | 106-50-3 | 0.160 | 0.076 | -0.001 | -0.176 | 108.16 | 0.48 ^c^ | 0 | 2 |
| 226 | 2-chloro-4-methylaniline | 615-65-6 | 0.180 | 0.571 | -0.015 | -0.195 | 141.61 | 2.25 ^c^ | 1 | 1 |
| 227 | 2-chloro-5-methylaniline | 95-81-8 | 0.200 | 0.525 | -0.013 | -0.200 | 141.61 | 2.25 ^c^ | 1 | 1 |
| 228 | 3-chloroaniline | 108-42-9 | 0.220 | 0.288 | -0.017 | -0.201 | 127.58 | 1.88 ^m^ | 1 | 1 |
| 229 | 4-propan-2-ylaniline | 99-88-7 | 0.220 | 0.491 | -0.003 | -0.197 | 135.23 | 2.31 ^m^ | 0 | 1 |
| 230 | 2,6-diethylaniline | 579-66-8 | 0.310 | 0.862 | 0.008 | -0.207 | 149.26 | 2.99 ^c^ | 0 | 1 |
| 231 | 2,4-dichloroaniline | 554-00-7 | 0.560 | 0.988 | -0.031 | -0.190 | 162.02 | 2.78 ^m^ | 2 | 1 |
| 232 | 2,5-dichloroaniline | 95-82-9 | 0.580 | 0.928 | -0.029 | -0.198 | 162.02 | 2.75 ^m^ | 2 | 1 |
| 233 | 4-butoxyaniline | 4344-55-2 | 0.610 | 0.678 | -0.004 | -0.184 | 165.26 | 2.22 ^c^ | 0 | 1 |
| 234 | ethyl 2-aminobenzoate | 87-25-2 | 0.650 | 0.845 | -0.041 | -0.170 | 165.21 | 2.57 ^m^ | 0 | 1 |
| 235 | 3,5-dichloroaniline | 626-43-7 | 0.710 | 0.977 | -0.030 | -0.200 | 162.02 | 2.90 ^m^ | 2 | 1 |
| 236 | 2,6-di(propan-2-yl)aniline | 24544-04-5 | 0.760 | 1.026 | 0.002 | -0.203 | 177.32 | 3.18 ^m^ | 0 | 1 |
| 237 | 4-pentyloxyaniline | 39905-50-5 | 0.970 | 0.923 | -0.004 | -0.184 | 179.29 | 2.61 ^c^ | 0 | 1 |
| 238 | 4-bromoaniline | 106-40-1 | 1.010 | 0.599 | -0.019 | -0.192 | 172.03 | 2.18 ^m^ | 1 | 1 |
| 239 | 2,4,6-trichloroaniline | 634-93-5 | 1.010 | 1.417 | -0.042 | -0.187 | 196.46 | 3.52 ^m^ | 3 | 1 |
| 240 | 4-butylaniline | 104-13-2 | 1.070 | 0.955 | -0.003 | -0.197 | 149.26 | 3.05 ^m^ | 0 | 1 |
| 241 | 2,4,5-trichloroaniline | 636-30-6 | 1.300 | 1.393 | -0.041 | -0.188 | 196.46 | 3.45 ^m^ | 3 | 1 |
| 242 | 4-hexyloxyaniline | 39905-57-2 | 1.380 | 1.014 | -0.007 | -0.194 | 193.32 | 3.01 ^c^ | 0 | 1 |
| 243 | 2,3,4,5-tetrachloroaniline | 634-83-3 | 1.960 | 1.680 | -0.047 | -0.187 | 230.90 | 4.04 ^m^ | 4 | 1 |
| 244 | phenylmethanol | 100-51-6 | -0.830 | -0.850 | -0.007 | -0.235 | 108.15 | 1.10 ^m^ | 0 | 1 |
| 245 | 1-phenylethanol | 98-85-1 | -0.660 | -0.638 | -0.008 | -0.236 | 122.18 | 1.42 ^m^ | 0 | 1 |
| 246 | N-phenylacetamide | 103-84-4 | -0.620 | -0.405 | -0.026 | -0.211 | 135.18 | 1.16 ^m^ | 0 | 1 |
| 247 | 2-phenylpropan-1-amine | 582-22-9 | -0.280 | -0.317 | -0.002 | -0.233 | 135.23 | 1.82 ^c^ | 0 | 1 |
| 248 | phenylmethanamine | 100-46-9 | -0.240 | -0.799 | -0.005 | -0.231 | 107.17 | 1.09 ^m^ | 0 | 1 |
| 249 | 2-phenylbut-3-yn-2-ol | 127-66-2 | -0.180 | -0.317 | -0.024 | -0.239 | 146.20 | 1.93 ^c^ | 0 | 1 |
| 250 | 1-phenylbutan-2-ol | 120055-09-6 | -0.160 | 0.152 | -0.003 | -0.238 | 150.24 | 2.65 ^c^ | 0 | 1 |
| 251 | ethynylbenzene | 536-74-3 | -0.140 | -0.075 | -0.061 | -0.215 | 102.14 | 2.16 ^m^ | 0 | 0 |
| 252 | chlorobenzene | 108-90-7 | -0.130 | 0.264 | -0.025 | -0.230 | 112.56 | 2.84 ^m^ | 1 | 0 |
| 253 | benzene | 71-43-2 | -0.120 | -0.564 | -0.009 | -0.247 | 78.12 | 2.13 ^m^ | 0 | 0 |
| 254 | methoxybenzene | 100-66-3 | -0.100 | -0.144 | -0.008 | -0.216 | 108.15 | 2.11 ^m^ | 0 | 0 |
| 255 | 1-phenylbutan-1-ol | 22144-60-1 | -0.010 | 0.254 | -0.013 | -0.235 | 150.24 | 2.79 ^c^ | 0 | 1 |
| 256 | 3-phenylbutan-1-ol | 2722-36-3 | 0.010 | 0.074 | -0.005 | -0.236 | 150.24 | 2.49 ^c^ | 0 | 1 |
| 257 | 2-phenylbutan-2-ol | 1565-75-9 | 0.060 | 0.046 | -0.006 | -0.237 | 150.24 | 2.47 ^c^ | 0 | 1 |
| 258 | (4-ethylphenyl)methanol | 768-59-2 | 0.070 | 0.107 | -0.006 | -0.227 | 136.21 | 2.38 ^c^ | 0 | 1 |
| 259 | chloromethylbenzene | 100-44-7 | 0.100 | 0.115 | -0.040 | -0.222 | 126.59 | 2.30 ^m^ | 1 | 0 |
| 260 | ethoxybenzene | 103-73-1 | 0.100 | 0.173 | -0.008 | -0.215 | 122.18 | 2.51 ^m^ | 0 | 0 |
| 261 | 2-(4-chlorophenyl)ethylamine | 156-41-2 | 0.140 | 0.140 | -0.025 | -0.221 | 155.64 | 1.93 ^c^ | 1 | 1 |
| 262 | (4-chlorophenyl)methanamine | 104-86-9 | 0.160 | -0.010 | -0.020 | -0.221 | 141.61 | 1.68 ^c^ | 1 | 1 |
| 263 | (4-propan-2-ylphenyl)methanol | 536-60-7 | 0.170 | 0.175 | -0.004 | -0.229 | 150.24 | 2.49 ^m^ | 0 | 1 |
| 264 | methylsulfanylbenzene | 100-68-5 | 0.180 | 0.384 | -0.028 | -0.203 | 124.21 | 2.74 ^m^ | 0 | 0 |
| 265 | methylbenzene | 108-88-3 | 0.250 | -0.048 | -0.007 | -0.237 | 92.15 | 2.69 ^m^ | 0 | 0 |
| 266 | 1-methoxy-4-methylbenzene | 104-93-8 | 0.250 | 0.413 | -0.007 | -0.209 | 122.18 | 2.81 ^m^ | 0 | 0 |
| 267 | 1-chloro-2-methoxybenzene | 766-51-8 | 0.270 | 0.403 | -0.021 | -0.210 | 142.59 | 2.68 ^m^ | 1 | 0 |
| 268 | 5-phenylpentan-1-ol | 10521-91-2 | 0.420 | 0.327 | -0.005 | -0.237 | 164.27 | 2.89 ^m^ | 0 | 1 |
| 269 | 2-bromoethylbenzene | 103-63-9 | 0.500 | 0.674 | -0.031 | -0.227 | 185.07 | 3.09 ^m^ | 1 | 0 |
| 270 | iodobenzene | 591-50-4 | 0.500 | 0.933 | -0.037 | -0.206 | 204.01 | 3.25 ^m^ | 1 | 0 |
| 271 | 1,2-dichlorobenzene | 95-50-1 | 0.530 | 0.780 | -0.036 | -0.224 | 147.00 | 3.38 ^m^ | 2 | 0 |
| 272 | 1-chloro-4-methoxybenzene | 623-12-1 | 0.600 | 0.488 | -0.024 | -0.204 | 142.59 | 2.78 ^m^ | 1 | 0 |
| 273 | naphthalen-1-yl acetate | 830-81-9 | 0.640 | 0.937 | -0.059 | -0.172 | 186.22 | 2.78 ^m^ | 0 | 0 |
| 274 | phenylmethyl 2-methylprop-2-enoate | 2495-37-6 | 0.650 | 0.378 | -0.048 | -0.204 | 176.23 | 2.16 ^m^ | 0 | 0 |
| 275 | 1,4-dibromobenzene | 106-37-6 | 0.680 | 1.196 | -0.039 | -0.211 | 235.90 | 3.79 ^m^ | 2 | 0 |
| 276 | propan-2-ylbenzene | 98-82-8 | 0.690 | 0.688 | -0.008 | -0.237 | 120.21 | 3.66 ^m^ | 0 | 0 |
| 277 | diethyl 2-(phenylmethyl)propanedioate | 607-81-8 | 0.710 | 0.516 | -0.010 | -0.236 | 250.32 | 2.76 ^m^ | 0 | 0 |
| 278 | 1,1-di(phenyl)propan-2-ol | 29338-49-6 | 0.750 | 1.023 | -0.022 | -0.225 | 212.31 | 3.80 ^c^ | 0 | 1 |
| 279 | 1,2-di(phenyl)propan-2-ol | 5342-87-0 | 0.800 | 0.990 | -0.016 | -0.224 | 212.31 | 3.69 ^c^ | 0 | 1 |
| 280 | 6-phenylhexan-1-ol | 2430-16-2 | 0.870 | 0.583 | -0.007 | -0.237 | 178.30 | 3.29 ^m^ | 0 | 1 |
| 281 | 1,3,5-trichlorobenzene | 108-70-3 | 0.870 | 1.297 | -0.050 | -0.225 | 181.44 | 4.15 ^m^ | 3 | 0 |
| 282 | (4-phenylphenyl)methanol | 3597-91-9 | 0.920 | 1.002 | -0.032 | -0.192 | 184.25 | 3.20 ^m^ | 0 | 1 |
| 283 | 1-bromo-4-methylbenzene | 106-38-7 | 1.000 | 0.777 | -0.023 | -0.219 | 171.04 | 3.31 ^c^ | 1 | 0 |
| 284 | phenylbenzene | 92-52-4 | 1.050 | 1.250 | -0.035 | -0.197 | 154.22 | 4.04 ^m^ | 0 | 0 |
| 285 | 1-bromo-4-ethylbenzene | 1585-07-5 | 1.100 | 1.023 | -0.023 | -0.219 | 185.07 | 3.70 ^c^ | 1 | 0 |
| 286 | 1,2,4-trichlorobenzene | 120-82-1 | 1.100 | 1.232 | -0.049 | -0.214 | 181.44 | 4.05 ^m^ | 3 | 0 |
| 287 | butylbenzene | 104-51-8 | 1.250 | 1.137 | -0.007 | -0.236 | 134.24 | 4.26 ^m^ | 0 | 0 |
| 288 | pentylbenzene | 538-68-1 | 1.790 | 1.597 | -0.007 | -0.236 | 148.27 | 4.90 ^m^ | 0 | 0 |
| 289 | 1-ethyl-4-phenylbenzene | 5707-44-8 | 1.970 | 1.714 | -0.032 | -0.192 | 182.28 | 4.60 ^c^ | 0 | 0 |
| 290 | 1,2,4,5-tetrachlorobenzene | 95-94-3 | 2.000 | 1.505 | -0.059 | -0.207 | 215.88 | 4.60 ^m^ | 4 | 0 |
| 291 | N-(4-formylphenyl)acetamide | 122-85-0 | -0.224 | 0.214 | -0.069 | -0.175 | 163.19 | 1.25 ^m^ | 0 | 1 |
| 292 | benzaldehyde | 100-52-7 | -0.196 | -0.198 | -0.071 | -0.192 | 106.13 | 1.48 ^m^ | 0 | 0 |
| 293 | 3,4,5-trihydroxybenzaldehyde | 13677-79-7 | -0.196 | 0.412 | -0.063 | -0.165 | 154.13 | 0.87^c^ | 0 | 3 |
| 294 | 3-hydroxy-4-methoxy-benzaldehyde | 621-59-0 | -0.141 | 0.086 | -0.065 | -0.171 | 152.16 | 0.97 ^m^ | 0 | 1 |
| 295 | 4-fluorobenzaldehyde | 459-57-4 | -0.127 | 0.022 | -0.074 | -0.194 | 124.12 | 1.51 ^m^ | 1 | 0 |
| 296 | 2,4,5-trimethoxybenzaldehyde | 4460-86-0 | -0.101 | 0.240 | -0.053 | -0.168 | 196.22 | 0.96 ^c^ | 0 | 0 |
| 297 | 4-methylbenzaldehyde | 104-87-0 | -0.057 | 0.185 | -0.067 | -0.192 | 120.16 | 2.19 ^c^ | 0 | 0 |
| 298 | 2,4-dimethoxybenzaldehyde | 613-45-6 | -0.056 | 0.091 | -0.053 | -0.179 | 166.19 | 1.22 ^c^ | 0 | 0 |
| 299 | 4-methoxybenzaldehyde | 123-11-5 | -0.047 | 0.082 | -0.060 | -0.181 | 136.16 | 1.61 ^m^ | 0 | 0 |
| 300 | 3-methoxy-4-hydroxybenzaldehyde | 121-33-5 | -0.030 | 0.171 | -0.059 | -0.171 | 152.16 | 1.21 ^m^ | 0 | 1 |
| 301 | 3-formylbenzonitrile | 24964-64-5 | -0.020 | 0.069 | -0.096 | -0.186 | 131.14 | 1.18 ^m^ | 0 | 0 |
| 302 | 2,3,4-trihydroxybenzaldehyde | 2144-08-3 | 0.001 | 0.246 | -0.056 | -0.176 | 154.13 | 0.87 ^c^ | 0 | 3 |
| 303 | 3-ethoxy-4-hydroxybenzaldehyde | 121-32-4 | 0.015 | 0.390 | -0.058 | -0.170 | 166.19 | 1.58 ^m^ | 0 | 1 |
| 304 | 4-formylbenzonitrile | 105-07-7 | 0.043 | 0.288 | -0.105 | -0.178 | 131.14 | 1.59 ^c^ | 0 | 0 |
| 305 | 4-ethoxybenzaldehyde | 10031-82-0 | 0.073 | 0.376 | -0.059 | -0.181 | 150.19 | 2.09 ^m^ | 0 | 0 |
| 306 | 2-fluorobenzaldehyde | 446-52-6 | 0.079 | 0.183 | -0.079 | -0.188 | 124.12 | 1.86 ^c^ | 1 | 0 |
| 307 | 3-methylbenzaldehyde | 620-23-5 | 0.081 | 0.195 | -0.069 | -0.191 | 120.16 | 2.19 ^c^ | 0 | 0 |
| 308 | 3-hydroxybenzaldehyde | 100-83-4 | 0.085 | 0.062 | -0.070 | -0.175 | 122.13 | 1.38 ^m^ | 0 | 1 |
| 309 | 2,4,6-trihydroxybenzaldehyde | 487-70-7 | 0.128 | 0.083 | -0.046 | -0.187 | 154.13 | 0.87 ^c^ | 0 | 3 |
| 310 | 3-nitrobenzaldehyde | 99-61-6 | 0.138 | 0.481 | -0.111 | -0.172 | 151.13 | 1.47 ^m^ | 0 | 0 |
| 311 | 2-methoxybenzaldehyde | 135-02-4 | 0.148 | 0.165 | -0.065 | -0.177 | 136.16 | 1.72 ^m^ | 0 | 0 |
| 312 | 3-fluorobenzaldehyde | 456-48-4 | 0.154 | 0.187 | -0.080 | -0.190 | 124.12 | 1.86 ^c^ | 1 | 0 |
| 313 | 2-chloro-6-nitrobenzaldehyde | 6361-22-4 | 0.155 | 0.949 | -0.118 | -0.159 | 185.57 | 2.19 ^c^ | 1 | 0 |
| 314 | 2-nitrobenzaldehyde | 552-89-6 | 0.167 | 0.579 | -0.113 | -0.164 | 151.13 | 1.74 ^m^ | 0 | 0 |
| 315 | 4-nitrobenzaldehyde | 555-16-8 | 0.203 | 0.597 | -0.121 | -0.164 | 151.13 | 1.56 ^m^ | 0 | 0 |
| 316 | 2-chloro-3-hydroxy-4-methoxybenzaldehyde | 37687-57-3 | 0.204 | 0.641 | -0.069 | -0.167 | 186.60 | 1.70 ^c^ | 1 | 1 |
| 317 | 4-dimethylaminobenzaldehyde | 100-10-7 | 0.231 | 0.280 | -0.051 | -0.173 | 149.21 | 1.81 ^m^ | 0 | 0 |
| 318 | 3-methoxybenzaldehyde | 591-31-1 | 0.232 | 0.187 | -0.067 | -0.173 | 136.16 | 1.71 ^m^ | 0 | 0 |
| 319 | 4-hydroxybenzaldehyde | 123-08-0 | 0.266 | -0.056 | -0.061 | -0.186 | 122.13 | 1.35 ^m^ | 0 | 1 |
| 320 | 3-hydroxy-4-nitrobenzaldehyde | 704-13-2 | 0.273 | 0.557 | -0.112 | -0.160 | 167.13 | 1.39 ^c^ | 0 | 1 |
| 321 | 4-ethylbenzaldehyde | 4748-78-1 | 0.291 | 0.451 | -0.067 | -0.192 | 134.19 | 2.59 ^c^ | 0 | 0 |
| 322 | 5-hydroxy-2-nitrobenzaldehyde | 42454-06-8 | 0.329 | 0.509 | -0.107 | -0.164 | 167.13 | 1.39 ^c^ | 0 | 1 |
| 323 | 2-hydroxy-3-methoxybenzaldehyde | 148-53-8 | 0.377 | 0.265 | -0.061 | -0.168 | 152.16 | 1.37 ^m^ | 0 | 1 |
| 324 | 4-chlorobenzaldehyde | 104-88-1 | 0.400 | 0.379 | -0.081 | -0.190 | 140.57 | 2.10 ^m^ | 1 | 0 |
| 325 | 3-chlorobenzaldehyde | 587-04-2 | 0.406 | 0.446 | -0.083 | -0.187 | 140.57 | 2.26 ^m^ | 1 | 0 |
| 326 | naphthalene-1-carbaldehyde | 66-77-3 | 0.423 | 0.805 | -0.082 | -0.155 | 156.19 | 2.72 ^c^ | 0 | 0 |
| 327 | 2-bromobenzaldehyde | 6630-33-7 | 0.477 | 0.793 | -0.082 | -0.184 | 185.02 | 2.51 ^c^ | 1 | 0 |
| 328 | 2-chlorobenzaldehyde | 89-98-5 | 0.487 | 0.470 | -0.083 | -0.186 | 140.57 | 2.33 ^m^ | 1 | 0 |
| 329 | 3-bromobenzaldehyde | 3132-99-8 | 0.506 | 0.800 | -0.083 | -0.182 | 185.02 | 2.51 ^c^ | 1 | 0 |
| 330 | 2,4-dihydroxybenzaldehyde | 95-01-2 | 0.515 | 0.076 | -0.057 | -0.183 | 138.13 | 1.15 ^c^ | 0 | 2 |
| 331 | 2-chloro-5-nitrobenzaldehyde | 6361-21-3 | 0.527 | 0.948 | -0.115 | -0.173 | 185.57 | 2.19 ^c^ | 1 | 0 |
| 332 | 4-bromobenzaldehyde | 1122-91-4 | 0.587 | 0.865 | -0.081 | -0.185 | 185.02 | 2.75 ^m^ | 1 | 0 |
| 333 | 4-hydroxy-3-nitrobenzaldehyde | 3011-34-5 | 0.614 | 0.426 | -0.099 | -0.175 | 167.13 | 1.48 ^m^ | 0 | 1 |
| 334 | 3-bromo-4-hydroxy-5-methoxy-benzaldehyde | 2973-76-4 | 0.617 | 0.831 | -0.068 | -0.171 | 231.05 | 1.98 ^c^ | 1 | 1 |
| 335 | 4,6-dimethoxy-2-hydroxybenzaldehyde | 708-76-9 | 0.617 | -0.004 | -0.043 | -0.184 | 182.19 | 0.93 ^c^ | 0 | 1 |
| 336 | 4-propan-2-ylbenzaldehyde | 122-03-2 | 0.670 | 0.683 | -0.067 | -0.192 | 148.22 | 2.92 ^c^ | 0 | 0 |
| 337 | 4-butoxybenzaldehyde | 5736-88-9 | 0.716 | 0.799 | -0.058 | -0.181 | 178.25 | 2.68 ^c^ | 0 | 0 |
| 338 | 2,3,4,5,6-pentafluorobenzaldehyde | 653-37-2 | 0.815 | 1.246 | -0.099 | -0.185 | 196.08 | 2.42 ^c^ | 5 | 0 |
| 339 | 2-hydroxy-3-nitrobenzaldehyde | 5274-70-4 | 0.870 | 0.644 | -0.121 | -0.152 | 167.13 | 1.39 ^c^ | 0 | 1 |
| 340 | 2-chloro-4-hydroxybenzaldehyde | 56962-11-9 | 0.890 | 0.514 | -0.072 | -0.185 | 156.57 | 1.96 ^c^ | 1 | 1 |
| 341 | 3,5-dibromo-4-hydroxybenzaldehyde | 2973-77-5 | 0.890 | 1.270 | -0.081 | -0.181 | 279.91 | 3.02 ^c^ | 2 | 1 |
| 342 | 5-chloro-2-hydroxybenzaldehyde | 635-93-8 | 1.009 | 0.609 | -0.079 | -0.170 | 156.57 | 1.96 ^c^ | 1 | 1 |
| 343 | 2,3-dihydroxybenzaldehyde | 24677-78-9 | 1.030 | 0.239 | -0.064 | -0.170 | 138.13 | 1.15 ^c^ | 0 | 2 |
| 344 | 2,4-dichlorobenzaldehyde | 874-42-0 | 1.036 | 0.866 | -0.091 | -0.185 | 175.01 | 2.76 ^c^ | 2 | 0 |
| 345 | 5-bromo-2-hydroxybenzaldehyde | 1761-61-1 | 1.107 | 0.956 | -0.079 | -0.167 | 201.02 | 2.50 ^m^ | 1 | 1 |
| 346 | 4-phenylbenzaldehyde | 3218-36-8 | 1.119 | 1.190 | -0.076 | -0.169 | 182.23 | 3.41 ^c^ | 0 | 0 |
| 347 | 4-methylnaphthalene-1-carboxaldehyde | 33738-48-6 | 1.123 | 1.074 | -0.079 | -0.153 | 170.22 | 3.19 ^c^ | 0 | 0 |
| 348 | 4-pentyloxybenzaldehyde | 5736-91-4 | 1.179 | 1.047 | -0.058 | -0.181 | 192.28 | 3.07 ^c^ | 0 | 0 |
| 349 | 2-methyl-1-naphthaldehyde | 35699-44-6 | 1.231 | 1.069 | -0.079 | -0.155 | 170.22 | 3.19 ^c^ | 0 | 0 |
| 350 | 6-chloro-2-fluoro-3-methylbenzaldehyde | 286474-59-7 | 1.238 | 0.824 | -0.083 | -0.180 | 172.59 | 2.85 ^c^ | 2 | 0 |
| 351 | 2-hydroxynaphthalene-1-carbaldehyde | 708-06-5 | 1.320 | 0.876 | -0.074 | -0.153 | 172.19 | 2.44 ^c^ | 0 | 1 |
| 352 | 2,3,5-trichlorobenzaldehyde | 56961-75-2 | 1.499 | 1.166 | -0.101 | -0.173 | 209.45 | 3.28 ^c^ | 3 | 0 |
| 353 | 9H-fluorene-2-carbaldehyde | 30084-90-3 | 1.499 | 1.306 | -0.075 | -0.160 | 194.24 | 3.44 ^c^ | 0 | 0 |
| 354 | 3,5-dibromo-2-hydroxybenzaldehyde | 90-59-5 | 1.638 | 1.353 | -0.088 | -0.165 | 279.91 | 3.02 ^c^ | 2 | 1 |
| 355 | phenanthrene-9-carbaldehyde | 4707-71-5 | 1.708 | 1.512 | -0.082 | -0.150 | 206.25 | 3.73 ^c^ | 0 | 0 |
| 356 | 3-chloro-2-fluoro-5-(trifluoromethyl)benzaldehyde | 261763-02-4 | 1.723 | 1.433 | -0.103 | -0.185 | 226.56 | 3.26 ^c^ | 5 | 0 |
| 357 | 5-nitropyrimidin-2-amine | 3073-77-6 | -0.430 | 0.169 | -0.085 | -0.163 | 139.13 | 1.07 ^c^ | 0 | 1 |
| 358 | (3-nitrophenyl)methanol | 619-25-0 | -0.220 | 0.209 | -0.092 | -0.178 | 153.15 | 1.21 ^m^ | 0 | 1 |
| 359 | 3-nitrobenzamide | 645-09-0 | -0.190 | 0.266 | -0.104 | -0.172 | 166.15 | 0.77 ^m^ | 0 | 1 |
| 360 | (2-nitrophenyl)methanol | 612-25-9 | -0.160 | 0.235 | -0.094 | -0.177 | 153.15 | 1.24 ^m^ | 0 | 1 |
| 361 | 3-nitropyridin-2-amine | 4214-75-9 | -0.010 | 0.306 | -0.097 | -0.148 | 139.13 | 1.07 ^c^ | 0 | 1 |
| 362 | 2-methoxy-5-nitropyridine | 5446-92-4 | -0.010 | 0.403 | -0.095 | -0.173 | 154.14 | 1.55 ^m^ | 0 | 0 |
| 363 | 1-methyl-2-nitrobenzene | 88-72-2 | 0.050 | 0.471 | -0.091 | -0.184 | 137.15 | 2.30 ^m^ | 0 | 0 |
| 364 | 1-methyl-3-nitrobenzene | 99-08-1 | 0.050 | 0.538 | -0.093 | -0.181 | 137.15 | 2.45 ^m^ | 0 | 0 |
| 365 | 8-nitroquinoline | 607-35-2 | 0.080 | 0.500 | -0.092 | -0.169 | 174.17 | 1.40 ^m^ | 0 | 0 |
| 366 | 2-nitroaniline | 88-74-4 | 0.080 | 0.544 | -0.087 | -0.143 | 138.14 | 1.83 ^m^ | 0 | 1 |
| 367 | 1-fluoro-4-nitrobenzene | 350-46-9 | 0.100 | 0.422 | -0.100 | -0.188 | 141.11 | 1.80 ^m^ | 1 | 0 |
| 368 | (4-nitrophenyl)methanol | 619-73-8 | 0.100 | 0.179 | -0.090 | -0.183 | 153.15 | 1.26 ^m^ | 0 | 1 |
| 369 | nitrobenzene | 98-95-3 | 0.140 | 0.218 | -0.096 | -0.191 | 123.12 | 1.85 ^m^ | 0 | 0 |
| 370 | 1-methyl-4-nitrobenzene | 99-99-0 | 0.170 | 0.496 | -0.092 | -0.186 | 137.15 | 2.37 ^m^ | 0 | 0 |
| 371 | 4-nitrobenzamide | 619-80-7 | 0.180 | 0.328 | -0.108 | -0.169 | 166.15 | 0.82 ^m^ | 0 | 1 |
| 372 | 1-fluoro-3-nitrobenzene | 402-67-5 | 0.200 | 0.475 | -0.104 | -0.180 | 141.11 | 1.90 ^m^ | 1 | 0 |
| 373 | 5-nitropyridin-2-amine | 4214-76-0 | 0.220 | 0.012 | -0.094 | -0.172 | 140.12 | 0.65 ^m^ | 0 | 1 |
| 374 | 4-fluoro-1-methyl-2-nitrobenzene | 446-10-6 | 0.250 | 0.728 | -0.099 | -0.174 | 155.14 | 2.61 ^c^ | 1 | 0 |
| 375 | 2-chloro-4-methyl-3-nitropyridine | 23056-39-5 | 0.290 | 0.813 | -0.097 | -0.187 | 172.58 | 2.51 ^c^ | 1 | 0 |
| 376 | 1,3-dimethyl-2-nitrobenzene | 81-20-9 | 0.300 | 0.789 | -0.084 | -0.181 | 151.18 | 2.95 ^m^ | 0 | 0 |
| 377 | 1-(3-nitrophenyl)ethanone | 121-89-1 | 0.320 | 0.547 | -0.106 | -0.169 | 165.16 | 1.42 ^m^ | 0 | 0 |
| 378 | 2-fluoro-1-methyl-4-nitrobenzene | 1427-07-2 | 0.330 | 0.733 | -0.099 | -0.179 | 155.14 | 2.61 ^c^ | 1 | 0 |
| 379 | 4-methyl-2-nitroaniline | 89-62-3 | 0.370 | 0.661 | -0.085 | -0.138 | 152.17 | 1.82 ^m^ | 0 | 1 |
| 380 | 4-methyl-5-nitropyridin-2-amine | 21901-40-6 | 0.370 | 0.396 | -0.082 | -0.163 | 153.16 | 1.54 ^c^ | 0 | 1 |
| 381 | 5-nitroquinoline | 607-34-1 | 0.390 | 0.761 | -0.106 | -0.160 | 174.17 | 1.86 ^m^ | 0 | 0 |
| 382 | methyl-4-nitrobenzoate | 619-50-1 | 0.400 | 0.833 | -0.110 | -0.183 | 181.16 | 1.94 ^m^ | 0 | 0 |
| 383 | 4-nitropyridine | 1122-61-8 | 0.410 | 0.028 | -0.114 | -0.175 | 124.11 | 0.60 ^m^ | 0 | 0 |
| 384 | 2-chloro-4-methyl-5-nitropyridine | 23056-33-9 | 0.420 | 0.905 | -0.110 | -0.182 | 172.58 | 2.51 ^c^ | 1 | 0 |
| 385 | 1-chloro-4-nitrobenzene | 100-00-5 | 0.430 | 0.729 | -0.105 | -0.180 | 157.56 | 2.39 ^m^ | 1 | 0 |
| 386 | 1,4-difluoro-2-nitrobenzene | 364-74-9 | 0.450 | 0.733 | -0.106 | -0.176 | 159.10 | 2.25 ^m^ | 2 | 0 |
| 387 | 6-nitroquinoline | 613-50-3 | 0.460 | 0.773 | -0.105 | -0.162 | 174.17 | 1.94 ^m^ | 0 | 0 |
| 388 | 2-methyl-4-nitroaniline | 99-52-5 | 0.490 | 0.480 | -0.076 | -0.157 | 152.17 | 1.68 ^c^ | 0 | 1 |
| 389 | 4-amino-3,5-dinitrobenzamide | 54321-79-8 | 0.510 | 0.760 | -0.125 | -0.139 | 226.17 | 0.00 ^c^ | 0 | 2 |
| 390 | 4-nitrobenzene-1,2-diamine | 99-56-9 | 0.520 | 0.552 | -0.077 | -0.145 | 153.16 | 1.09 ^m^ | 0 | 2 |
| 391 | (3,5-dinitrophenyl)methanol | 71022-43-0 | 0.530 | 0.666 | -0.115 | -0.179 | 198.15 | 1.42 ^c^ | 0 | 1 |
| 392 | 2,4-dinitroaniline | 97-02-9 | 0.530 | 0.816 | -0.109 | -0.151 | 183.14 | 1.90 ^m^ | 0 | 1 |
| 393 | 1,2-dimethyl-3-nitrobenzene | 83-41-0 | 0.560 | 0.755 | -0.086 | -0.179 | 151.18 | 2.83 ^m^ | 0 | 0 |
| 394 | 1,2-dimethyl-4-nitrobenzene | 99-51-4 | 0.590 | 0.791 | -0.089 | -0.180 | 151.18 | 2.91 ^m^ | 0 | 0 |
| 395 | 1-methoxy-3-nitrobenzene | 555-03-3 | 0.670 | 0.610 | -0.092 | -0.159 | 153.15 | 2.16 ^m^ | 0 | 0 |
| 396 | 1-chloro-2-methyl-3-nitrobenzene | 83-42-1 | 0.680 | 0.968 | -0.098 | -0.176 | 171.59 | 3.09 ^m^ | 1 | 0 |
| 397 | 1-chloro-2-nitrobenzene | 88-73-3 | 0.680 | 0.715 | -0.097 | -0.182 | 157.56 | 2.52 ^m^ | 1 | 0 |
| 398 | N-[(4-nitrophenyl)methylidene]hydroxylamine | 1129-37-9 | 0.680 | 0.677 | -0.107 | -0.159 | 166.15 | 1.95 ^m^ | 0 | 1 |
| 399 | 1-chloro-3-nitrobenzene | 121-73-3 | 0.730 | 0.749 | -0.106 | -0.176 | 157.56 | 2.46 ^m^ | 1 | 0 |
| 400 | 1-bromo-2-nitrobenzene | 577-19-5 | 0.750 | 0.988 | -0.096 | -0.177 | 202.01 | 2.52 ^m^ | 1 | 0 |
| 401 | 2-chloro-4-nitroaniline | 121-87-9 | 0.750 | 0.807 | -0.087 | -0.156 | 172.58 | 2.14 ^m^ | 1 | 1 |
| 402 | 4,5-difluoro-2-nitroaniline | 78056-39-0 | 0.750 | 0.843 | -0.098 | -0.142 | 174.12 | 1.50 ^c^ | 2 | 1 |
| 403 | 4-ethoxy-2-nitroaniline | 616-86-4 | 0.760 | 0.792 | -0.086 | -0.124 | 182.20 | 1.31 ^c^ | 0 | 1 |
| 404 | 2-chloro-1-fluoro-4-nitrobenzene | 350-30-1 | 0.800 | 0.945 | -0.108 | -0.179 | 175.55 | 2.66 ^c^ | 2 | 0 |
| 405 | 2-chloro-5-nitropyridine | 4548-45-2 | 0.800 | 0.744 | -0.116 | -0.183 | 158.55 | 2.04 ^c^ | 1 | 0 |
| 406 | 4-chloro-1-methyl-2-nitrobenzene | 89-59-8 | 0.820 | 0.964 | -0.101 | -0.171 | 171.59 | 3.05 ^m^ | 1 | 0 |
| 407 | methyl-4-chloro-2-nitrobenzoate | 42087-80-9 | 0.820 | 1.073 | -0.105 | -0.177 | 215.60 | 2.25 ^c^ | 1 | 0 |
| 408 | 1-ethoxy-4-nitrobenzene | 100-29-8 | 0.830 | 0.788 | -0.085 | -0.169 | 167.18 | 2.53 ^m^ | 0 | 0 |
| 409 | 2,6-dinitroaniline | 606-22-4 | 0.840 | 0.891 | -0.120 | -0.138 | 183.14 | 1.79 ^m^ | 0 | 1 |
| 410 | 1,3,5-trimethyl-2-nitrobenzene | 603-71-4 | 0.860 | 1.052 | -0.081 | -0.179 | 165.21 | 3.40 ^c^ | 0 | 0 |
| 411 | 2-chloro-3-nitropyridine | 5470-18-8 | 0.870 | 0.675 | -0.108 | -0.185 | 158.55 | 2.04 ^c^ | 1 | 0 |
| 412 | 1,3-dinitrobenzene | 99-65-0 | 0.890 | 0.705 | -0.121 | -0.195 | 168.12 | 1.49 ^m^ | 0 | 0 |
| 413 | 6-nitroquinolin-5-amine | 35975-00-9 | 0.920 | 0.832 | -0.094 | -0.136 | 189.19 | 1.61 ^m^ | 0 | 1 |
| 414 | 3,5-dinitroaniline | 618-87-1 | 0.940 | 0.879 | -0.114 | -0.141 | 183.14 | 1.89 ^m^ | 0 | 1 |
| 415 | 2,4-dichloro-1-nitrobenzene | 611-06-3 | 0.990 | 1.115 | -0.105 | -0.181 | 192.00 | 3.09 ^m^ | 2 | 0 |
| 416 | 1-nitronaphthalene | 86-57-7 | 1.000 | 1.112 | -0.097 | -0.149 | 173.18 | 3.19 ^m^ | 0 | 0 |
| 417 | 2-methyl-1-nitronaphthalene | 881-03-8 | 1.040 | 1.293 | -0.088 | -0.151 | 187.21 | 3.47 ^c^ | 0 | 0 |
| 418 | 1,2-dichloro-3-nitrobenzene | 3209-22-1 | 1.070 | 1.078 | -0.102 | -0.179 | 192.00 | 3.05 ^m^ | 2 | 0 |
| 419 | 2-bromo-5-nitropyridine | 4487-59-6 | 1.070 | 1.112 | -0.116 | -0.174 | 203.00 | 2.34 ^c^ | 1 | 0 |
| 420 | 1-fluoro-3-iodo-5-nitrobenzene | 3819-88-3 | 1.090 | 1.523 | -0.112 | -0.159 | 267.00 | 3.40 ^c^ | 2 | 0 |
| 421 | (3,4-dinitrophenyl)methanol | 79544-31-3 | 1.090 | 0.652 | -0.111 | -0.176 | 198.15 | 1.42 ^c^ | 0 | 1 |
| 422 | 2-chloro-4,6-dinitroaniline | 3531-19-9 | 1.120 | 1.064 | -0.118 | -0.148 | 217.58 | 1.69 ^c^ | 1 | 1 |
| 423 | 1,4-dichloro-2-nitrobenzene | 89-61-2 | 1.130 | 1.074 | -0.106 | -0.172 | 192.00 | 3.03 ^m^ | 2 | 0 |
| 424 | 1,3-dichloro-5-nitrobenzene | 618-62-2 | 1.130 | 1.147 | -0.115 | -0.172 | 192.00 | 3.09 ^m^ | 2 | 0 |
| 425 | 1-bromo-2-methyl-4-nitrobenzene | 7149-70-4 | 1.160 | 1.302 | -0.101 | -0.173 | 216.04 | 3.26 ^c^ | 1 | 0 |
| 426 | 1,2-dichloro-4-nitrobenzene | 99-54-7 | 1.160 | 1.125 | -0.112 | -0.174 | 192.00 | 3.04 ^m^ | 2 | 0 |
| 427 | 1-(chloromethyl)-4-nitrobenzene | 100-14-1 | 1.180 | 0.900 | -0.106 | -0.187 | 171.59 | 2.61 ^c^ | 1 | 0 |
| 428 | 2-bromo-4,6-dinitroaniline | 1817-73-8 | 1.240 | 1.307 | -0.117 | -0.147 | 262.03 | 1.96 ^c^ | 1 | 1 |
| 429 | 2,4-dichloro-6-nitroaniline | 2683-43-4 | 1.260 | 1.131 | -0.107 | -0.135 | 207.02 | 2.25 ^c^ | 2 | 1 |
| 430 | 1,4-dinitrobenzene | 100-25-4 | 1.300 | 0.861 | -0.134 | -0.180 | 168.12 | 1.46 ^m^ | 0 | 0 |
| 431 | 1-nitro-2-phenylbenzene | 86-00-0 | 1.300 | 1.419 | -0.087 | -0.167 | 199.22 | 3.69 ^c^ | 0 | 0 |
| 432 | 2-chloro-6-methoxy-3-nitropyridine | 38533-61-8 | 1.360 | 0.776 | -0.097 | -0.176 | 188.58 | 2.02 ^c^ | 1 | 0 |
| 433 | 1,4-dibromo-2-nitrobenzene | 3460-18-2 | 1.370 | 1.584 | -0.105 | -0.165 | 280.90 | 3.58 ^c^ | 2 | 0 |
| 434 | 1-butoxy-4-nitrobenzene | 7244-78-2 | 1.420 | 1.317 | -0.084 | -0.169 | 195.24 | 3.50 ^m^ | 0 | 0 |
| 435 | 1,2,3-trichloro-4-nitrobenzene | 17700-09-3 | 1.510 | 1.377 | -0.108 | -0.179 | 226.44 | 3.61 ^m^ | 3 | 0 |
| 436 | 4-methyl-1,2-dinitrobenzene | 610-39-9 | 1.520 | 0.904 | -0.112 | -0.179 | 182.15 | 2.08 ^m^ | 0 | 0 |
| 437 | 1,2,4-trichloro-5-nitrobenzene | 89-69-0 | 1.530 | 1.334 | -0.112 | -0.171 | 226.44 | 3.48 ^m^ | 3 | 0 |
| 438 | 1-nitro-3-phenylbenzene | 2113-58-8 | 1.570 | 1.446 | -0.095 | -0.157 | 199.22 | 3.69 ^m^ | 0 | 0 |
| 439 | 2,4-dibromo-6-nitroaniline | 827-23-6 | 1.620 | 1.482 | -0.105 | -0.134 | 295.92 | 2.80 ^c^ | 2 | 1 |
| 440 | 4,5-dichloro-2-nitroaniline | 6641-64-1 | 1.660 | 1.121 | -0.104 | -0.138 | 207.02 | 2.25 ^c^ | 2 | 1 |
| 441 | 1-fluoro-2,4-dinitrobenzene | 70-34-8 | 1.710 | 0.902 | -0.122 | -0.191 | 186.11 | 1.50 ^m^ | 1 | 0 |
| 442 | 1,2,3,4-tetrachloro-5-nitrobenzene | 879-39-0 | 1.780 | 1.505 | -0.115 | -0.171 | 260.88 | 3.93 ^m^ | 4 | 0 |
| 443 | 1,2,4,5-tetrachloro-3-nitrobenzene | 117-18-0 | 1.820 | 1.472 | -0.100 | -0.182 | 260.88 | 3.89 ^m^ | 4 | 0 |
| 444 | 1,2,3,5-tetrafluoro-4-nitrobenzene | 314-41-0 | 1.870 | 1.195 | -0.107 | -0.185 | 195.08 | 2.56 ^c^ | 4 | 0 |
| 445 | 4-nitroaniline | 100-01-6 | 1.880 | 1.011 | -0.108 | -0.187 | 177.09 | 2.42 ^c^ | 3 | 0 |
| 446 | 4-nitro-N-phenylaniline | 836-30-6 | 1.890 | 1.564 | -0.082 | -0.140 | 214.24 | 3.74 ^m^ | 0 | 1 |
| 447 | 2,6-dichloro-3-nitropyridine | 16013-85-7 | 2.030 | 0.987 | -0.116 | -0.179 | 192.99 | 1.96 ^m^ | 2 | 0 |
| 448 | 1,5-difluoro-2,4-dinitrobenzene | 327-92-4 | 2.080 | 1.230 | -0.123 | -0.191 | 204.10 | 2.23 ^c^ | 2 | 0 |
| 449 | 1-iodo-2,4-dinitrobenzene | 709-49-9 | 2.120 | 1.893 | -0.123 | -0.160 | 294.01 | 3.21 ^c^ | 1 | 0 |
| 450 | 1,3,5-trichloro-2,4-dinitrobenzene | 6284-83-9 | 2.190 | 1.606 | -0.111 | -0.188 | 271.44 | 3.51 ^c^ | 3 | 0 |
| 451 | 1,2-dichloro-4,5-dinitrobenzene | 6306-39-4 | 2.210 | 1.517 | -0.130 | -0.170 | 237.00 | 2.99 ^c^ | 2 | 0 |
| 452 | 1-bromo-2,4-dinitrobenzene | 584-48-5 | 2.310 | 1.564 | -0.124 | -0.174 | 247.01 | 2.75 ^c^ | 1 | 0 |
| 453 | 1,2,3,4,5-pentafluoro-6-nitrobenzene | 880-78-4 | 2.430 | 1.455 | -0.114 | -0.183 | 213.07 | 2.70 ^c^ | 5 | 0 |
| 454 | 1,2,4-trichloro-3,5-dinitrobenzene | 2678-21-9 | 2.590 | 1.660 | -0.126 | -0.172 | 271.44 | 3.51 ^c^ | 3 | 0 |
| 455 | 1,2,4,5-tetrachloro-3,6-dinitrobenzene | 20098-38-8 | 2.740 | 1.713 | -0.112 | -0.185 | 305.88 | 4.03 ^c^ | 4 | 0 |
| 456 | pyridine-4-carbonitrile | 100-48-1 | -0.820 | -0.570 | -0.084 | -0.202 | 104.12 | 0.46 ^m^ | 0 | 0 |
| 457 | pyridine-2-carbonitrile | 100-70-9 | -0.790 | -0.725 | -0.076 | -0.213 | 104.12 | 0.40 ^m^ | 0 | 0 |
| 458 | pyridine-3-carbonitrile | 100-54-9 | -0.740 | -0.726 | -0.076 | -0.210 | 104.12 | 0.36 ^m^ | 0 | 0 |
| 459 | benzonitrile | 100-47-0 | -0.520 | -0.347 | -0.061 | -0.215 | 103.13 | 1.56 ^m^ | 0 | 0 |
| 460 | 2-aminobenzonitrile | 1885-29-6 | -0.500 | 0.037 | -0.050 | -0.177 | 118.15 | 1.40 ^m^ | 0 | 1 |
| 461 | 3-aminobenzonitrile | 2237-30-1 | -0.470 | -0.108 | -0.053 | -0.177 | 118.15 | 1.07 ^m^ | 0 | 1 |
| 462 | 4-cyanobenzamide | 3034-34-2 | -0.380 | -0.216 | -0.084 | -0.190 | 146.16 | 0.48 ^m^ | 0 | 1 |
| 463 | 2-(4-hydroxyphenyl)acetonitrile | 14191-95-8 | -0.380 | -0.172 | -0.028 | -0.209 | 133.16 | 1.56 ^c^ | 0 | 1 |
| 464 | 4-acetylbenzonitrile | 1443-80-7 | -0.370 | 0.216 | -0.096 | -0.179 | 145.17 | 1.22 ^m^ | 0 | 0 |
| 465 | 2-cyanobenzamide | 17174-98-0 | -0.320 | -0.150 | -0.082 | -0.192 | 146.16 | 0.75 ^c^ | 0 | 1 |
| 466 | 4-fluorobenzonitrile | 1194-02-1 | -0.260 | 0.103 | -0.062 | -0.212 | 121.12 | 2.05 ^c^ | 1 | 0 |
| 467 | 3-methylbenzonitrile | 620-22-4 | -0.250 | 0.149 | -0.059 | -0.209 | 117.16 | 2.38 ^c^ | 0 | 0 |
| 468 | 2-methylbenzonitrile | 529-19-1 | -0.240 | 0.070 | -0.059 | -0.209 | 117.16 | 2.21 ^m^ | 0 | 0 |
| 469 | 4-methylbenzonitrile | 104-85-8 | -0.100 | 0.140 | -0.056 | -0.209 | 117.16 | 2.38 ^c^ | 0 | 0 |
| 470 | 3-chlorobenzonitrile | 766-84-7 | -0.060 | 0.424 | -0.074 | -0.204 | 137.57 | 2.43 ^c^ | 1 | 0 |
| 471 | 4-hydroxy-3-methoxybenzonitrile | 4421-08-3 | -0.030 | -0.224 | -0.046 | -0.189 | 149.16 | 0.82 ^m^ | 0 | 1 |
| 472 | 4-chlorobenzonitrile | 623-03-0 | 0.000 | 0.346 | -0.072 | -0.202 | 137.57 | 2.24 ^m^ | 1 | 0 |
| 473 | 3-methoxybenzonitrile | 1527-89-5 | 0.050 | 0.015 | -0.057 | -0.191 | 133.16 | 1.66 ^c^ | 0 | 0 |
| 474 | 4-methoxybenzonitrile | 874-90-8 | 0.100 | -0.153 | -0.047 | -0.199 | 133.16 | 1.49 ^m^ | 0 | 0 |
| 475 | 4-aminobenzonitrile | 873-74-5 | 0.240 | -0.218 | -0.038 | -0.190 | 118.15 | 1.13 ^c^ | 0 | 1 |
| 476 | 2-chlorobenzonitrile | 873-32-5 | 0.280 | 0.414 | -0.072 | -0.204 | 137.57 | 2.43 ^c^ | 1 | 0 |
| 477 | ethyl-4-cyanobenzoate | 7153-22-2 | 0.370 | 0.587 | -0.088 | -0.194 | 175.20 | 1.98 ^c^ | 0 | 0 |
| 478 | 2-amino-5-chlorobenzonitrile | 5922-60-1 | 0.440 | 0.484 | -0.063 | -0.169 | 152.59 | 1.65 ^c^ | 1 | 1 |
| 479 | 3-nitrobenzonitrile | 619-24-9 | 0.450 | 0.402 | -0.116 | -0.186 | 148.13 | 1.17 ^m^ | 0 | 0 |
| 480 | 4-nitrobenzonitrile | 619-72-7 | 0.570 | 0.489 | -0.123 | -0.182 | 148.13 | 1.19 ^m^ | 0 | 0 |
| 481 | naphthalene-1-carbonitrile | 86-53-3 | 0.690 | 0.822 | -0.078 | -0.164 | 153.19 | 2.91 ^c^ | 0 | 0 |
| 482 | 2-nitrobenzonitrile | 612-24-8 | 1.080 | 0.427 | -0.118 | -0.181 | 148.13 | 1.16 ^m^ | 0 | 0 |
| 483 | 4-phenylbenzonitrile | 2920-38-9 | 1.240 | 1.210 | -0.070 | -0.179 | 179.23 | 3.60 ^c^ | 0 | 0 |
| 484 | 4-chloro-3-nitrobenzonitrile | 939-80-0 | 1.710 | 0.989 | -0.116 | -0.179 | 182.57 | 2.38 ^c^ | 1 | 0 |
| 485 | 3-hydroxybenzoic acid | 99-06-9 | -0.814 | 0.217 | -0.058 | -0.183 | 138.13 | 1.50 ^m^ | 0 | 2 |
| 486 | 2-hydroxybenzoic acid | 69-72-7 | -0.512 | 0.466 | -0.052 | -0.190 | 138.13 | 2.26 ^m^ | 0 | 2 |
| 487 | 4-phenylbenzoic acid | 92-92-2 | 0.980 | 1.304 | -0.065 | -0.178 | 198.23 | 3.75 ^m^ | 0 | 1 |
| 488 | 1-phenylethanone | 98-86-2 | -0.050 | -0.074 | -0.063 | -0.193 | 120.16 | 1.63 ^m^ | 0 | 0 |
| 489 | 1-phenylpropan-1-one | 93-55-0 | 0.050 | 0.261 | -0.062 | -0.193 | 134.19 | 2.19 ^m^ | 0 | 0 |
| 490 | 2-hydroxy-1-phenylethanone | 582-24-1 | 0.078 | 0.195 | -0.065 | -0.192 | 136.16 | 1.92 ^m^ | 0 | 1 |
| 491 | benzenecarbothioamide | 2227-79-4 | 0.090 | 0.416 | -0.067 | -0.148 | 137.21 | 1.49 ^m^ | 0 | 1 |
| 492 | 1-phenylbutan-1-one | 495-40-9 | 0.300 | 0.605 | -0.062 | -0.193 | 148.22 | 2.77 ^m^ | 0 | 0 |
| 493 | 1-phenylpentan-1-one | 1009-14-9 | 0.560 | 0.695 | -0.062 | -0.193 | 162.25 | 2.78 ^c^ | 0 | 0 |
| 494 | di(phenyl)methanone | 119-61-9 | 0.870 | 1.054 | -0.071 | -0.181 | 182.23 | 3.18 ^m^ | 0 | 0 |
| 495 | (4-bromophenyl)-phenylmethanone | 90-90-4 | 1.260 | 1.661 | -0.079 | -0.177 | 261.12 | 4.06 ^c^ | 1 | 0 |
| 496 | isothiocyanatobenzene | 103-72-0 | 1.410 | 0.835 | -0.056 | -0.182 | 135.19 | 3.37 ^m^ | 0 | 0 |
| 497 | (4-chlorophenyl)-phenylmethanone | 134-85-0 | 1.500 | 1.370 | -0.079 | -0.179 | 216.67 | 3.79 ^c^ | 1 | 0 |
| 498 | 2-methylnaphthalene-1,4-dione | 58-27-5 | 1.540 | 0.924 | -0.119 | -0.149 | 172.19 | 2.20 ^m^ | 0 | 0 |
| 499 | 1-phenylheptan-1-one | 1671-75-6 | 1.560 | 1.206 | -0.062 | -0.192 | 190.31 | 3.57 ^c^ | 0 | 0 |
| 500 | 1-phenyloctan-1-one | 1674-37-9 | 1.890 | 1.450 | -0.061 | -0.193 | 204.34 | 3.97 ^c^ | 0 | 0 |
| *Compounds for QSAR validation* | | | | | | | | | | |
| 501 | 3-(hydroxymethyl)phenol | 620-24-6 | -1.043 | -0.657 | -0.009 | -0.214 | 124.15 | 0.49 ^m^ | 0 | 2 |
| 502 | 4-hydroxybenzamide | 619-57-8 | -0.780 | -0.562 | -0.035 | -0.203 | 137.15 | 0.33 ^m^ | 0 | 2 |
| 503 | 4-(4-hydroxyphenyl)butan-2-one | 5471-51-2 | -0.500 | 0.413 | -0.021 | -0.201 | 164.22 | 2.34 ^c^ | 0 | 1 |
| 504 | 3-methoxyphenol | 150-19-6 | -0.326 | -0.266 | -0.004 | -0.215 | 124.15 | 1.58 ^m^ | 0 | 1 |
| 505 | 4-methylphenol | 106-44-5 | -0.184 | -0.052 | -0.010 | -0.209 | 108.15 | 1.94 ^m^ | 0 | 1 |
| 506 | 4-aminophenol | 123-30-8 | -0.076 | -0.504 | -0.007 | -0.184 | 109.14 | 0.04 ^m^ | 0 | 2 |
| 507 | 2-hydroxybenzonitrile | 611-20-1 | 0.034 | -0.014 | -0.057 | -0.196 | 119.13 | 1.61 ^m^ | 0 | 1 |
| 508 | 3,4-dimethylphenol | 95-65-8 | 0.122 | 0.136 | -0.004 | -0.212 | 122.18 | 2.23 ^m^ | 0 | 1 |
| 509 | 2-chlorophenol | 95-57-8 | 0.183 | 0.315 | -0.025 | -0.214 | 128.56 | 2.15 ^m^ | 1 | 1 |
| 510 | 2,3,6-trimethylphenol | 2416-94-6 | 0.277 | 0.420 | 0.000 | -0.215 | 136.21 | 2.67 ^m^ | 0 | 1 |
| 511 | 2,3,5-trimethylphenol | 697-82-5 | 0.360 | 0.717 | -0.002 | -0.215 | 136.21 | 3.16 ^c^ | 0 | 1 |
| 512 | 2-methoxy-4-prop-2-enylphenol | 97-53-0 | 0.420 | 0.373 | -0.005 | -0.202 | 164.22 | 2.27 ^m^ | 0 | 1 |
| 513 | ethyl-3-hydroxybenzoate | 7781-98-8 | 0.478 | 0.607 | -0.052 | -0.185 | 166.19 | 2.47 ^m^ | 0 | 1 |
| 514 | 4-chlorophenol | 106-48-9 | 0.545 | 0.489 | -0.027 | -0.205 | 128.56 | 2.39 ^m^ | 1 | 1 |
| 515 | 3-methyl-2-nitrophenol | 4920-77-8 | 0.610 | 0.695 | -0.103 | -0.147 | 153.15 | 2.29 ^m^ | 0 | 1 |
| 516 | 2-methyl-5-nitrophenol | 5428-54-6 | 0.660 | 0.604 | -0.092 | -0.160 | 153.15 | 2.18 ^c^ | 0 | 1 |
| 517 | 3-tert-butylphenol | 585-34-2 | 0.730 | 0.665 | -0.009 | -0.216 | 150.24 | 3.05 ^m^ | 0 | 1 |
| 518 | 2-amino-4-chlorophenol | 95-85-2 | 0.775 | 0.641 | -0.011 | -0.195 | 143.58 | 1.81 ^m^ | 1 | 2 |
| 519 | benzene-1,2,3-triol | 87-66-1 | 0.850 | -0.288 | 0.007 | -0.222 | 126.12 | 1.00 ^m^ | 0 | 3 |
| 520 | 6-amino-2,4-dimethylphenol | 41458-65-5 | 0.886 | 0.348 | -0.005 | -0.203 | 137.20 | 1.91 ^c^ | 0 | 2 |
| 521 | 2-butan-2-ylphenol | 89-72-5 | 0.980 | 0.813 | -0.010 | -0.214 | 150.24 | 3.27 ^m^ | 0 | 1 |
| 522 | 2,4,6-tribromobenzene-1,3-diol | 2437-49-2 | 1.060 | 1.938 | -0.058 | -0.180 | 346.79 | 4.37 ^m^ | 3 | 2 |
| 523 | (3-hydroxyphenyl) benzoate | 136-36-7 | 1.110 | 1.166 | -0.059 | -0.173 | 214.23 | 3.17 ^c^ | 0 | 1 |
| 524 | 2,3,5,6-tetrafluorophenol | 769-39-1 | 1.167 | 1.045 | -0.029 | -0.231 | 166.08 | 2.32 ^c^ | 4 | 1 |
| 525 | 4-methyl-2,6-dinitrophenol | 609-93-8 | 1.230 | 0.943 | -0.126 | -0.147 | 198.15 | 2.14 ^c^ | 0 | 1 |
| 526 | 2,3-dichlorophenol | 576-24-9 | 1.276 | 0.937 | -0.034 | -0.212 | 163.00 | 2.84 ^m^ | 2 | 1 |
| 527 | 4-amino-3-methylphenol | 2835-99-6 | 1.310 | 0.288 | -0.002 | -0.187 | 123.17 | 1.45 ^c^ | 0 | 2 |
| 528 | (2,4-dihydroxyphenyl)-phenylmethanone | 131-56-6 | 1.370 | 1.024 | -0.071 | -0.163 | 214.23 | 2.70 ^c^ | 0 | 2 |
| 529 | 4-nitrophenol | 100-02-7 | 1.420 | 0.389 | -0.088 | -0.174 | 139.12 | 1.91 ^m^ | 0 | 1 |
| 530 | 4-cyclohexylphenol | 1131-60-8 | 1.560 | 1.511 | -0.009 | -0.210 | 176.28 | 4.22 ^m^ | 0 | 1 |
| 531 | 4-nitro-3-(trifluoromethyl)phenol | 88-30-2 | 1.650 | 1.452 | -0.098 | -0.175 | 207.12 | 2.60 ^c^ | 3 | 1 |
| 532 | 2,4-dichloro-6-nitrophenol | 609-89-2 | 1.745 | 1.410 | -0.125 | -0.139 | 208.00 | 2.75 ^c^ | 2 | 1 |
| 533 | 4-chloro-5-methyl-2-propan-2-ylphenol | 89-68-9 | 1.854 | 1.420 | -0.016 | -0.207 | 184.68 | 3.92 ^m^ | 1 | 1 |
| 534 | 4-chloro-2-nitrophenol | 89-64-5 | 2.053 | 1.045 | -0.116 | -0.143 | 173.56 | 2.48 ^m^ | 1 | 1 |
| 535 | 4-(4-bromophenyl)phenol | 29558-77-8 | 2.310 | 1.809 | -0.039 | -0.182 | 249.11 | 4.24 ^c^ | 1 | 1 |
| 536 | 2,3,4,5,6-pentabromophenol | 608-71-9 | 2.664 | 1.955 | -0.086 | -0.165 | 488.57 | 5.30 ^m^ | 5 | 1 |
| 537 | 2-ethylaniline | 578-54-1 | -0.220 | -0.062 | -0.001 | -0.203 | 121.20 | 1.74 ^m^ | 0 | 1 |
| 538 | 4-chloroaniline | 106-47-8 | 0.050 | 0.294 | -0.019 | -0.193 | 127.58 | 1.83 ^m^ | 1 | 1 |
| 539 | 2,6-dichloroaniline | 608-31-1 | 0.330 | 0.953 | -0.028 | -0.197 | 162.02 | 2.76 ^m^ | 2 | 1 |
| 540 | 4-fluoro-3-(trifluoromethyl)aniline | 2357-47-3 | 0.770 | 1.174 | -0.041 | -0.183 | 179.13 | 2.29 ^c^ | 4 | 1 |
| 541 | 2,3,5,6-tetrachloroaniline | 3481-20-7 | 1.760 | 1.768 | -0.046 | -0.193 | 230.90 | 4.10 ^m^ | 4 | 1 |
| 542 | 3-phenylpropan-1-ol | 122-97-4 | -0.210 | -0.255 | -0.005 | -0.236 | 136.21 | 1.88 ^m^ | 0 | 1 |
| 543 | 2-methyl-4-phenylbutan-2-ol | 103-05-9 | -0.070 | 0.295 | -0.005 | -0.235 | 164.27 | 2.65 ^c^ | 0 | 1 |
| 544 | 4-phenylbutan-1-ol | 3360-41-6 | 0.120 | 0.061 | -0.008 | -0.237 | 150.24 | 2.35 ^m^ | 0 | 1 |
| 545 | 1,4-dimethylbenzene | 106-42-3 | 0.250 | 0.109 | -0.006 | -0.228 | 106.18 | 3.15 ^m^ | 0 | 0 |
| 546 | 4-phenylbutan-1-amine | 13214-66-9 | 0.620 | 0.150 | -0.007 | -0.230 | 149.26 | 2.40 ^m^ | 0 | 1 |
| 547 | bromobenzene | 108-86-1 | 0.750 | 0.462 | -0.025 | -0.226 | 157.01 | 2.99 ^m^ | 1 | 0 |
| 548 | 1,2-dichloro-4-methylbenzene | 95-75-0 | 1.070 | 1.061 | -0.034 | -0.218 | 161.03 | 4.05 ^m^ | 2 | 0 |
| 549 | 3,4-dimethoxy-5-hydroxybenzaldehyde | 29865-90-5 | -0.390 | 0.126 | -0.064 | -0.171 | 182.19 | 0.93 ^c^ | 0 | 1 |
| 550 | terephthalaldehyde | 623-27-8 | -0.086 | 0.207 | -0.106 | -0.169 | 134.14 | 1.40 ^c^ | 0 | 0 |
| 551 | 2-methylbenzaldehyde | 529-20-4 | 0.011 | 0.117 | -0.071 | -0.188 | 120.16 | 2.26 ^m^ | 0 | 0 |
| 552 | 3,4-dihydroxybenzaldehyde | 139-85-5 | 0.107 | 0.147 | -0.061 | -0.172 | 138.13 | 1.09 ^m^ | 0 | 2 |
| 553 | benzene-1,3-dicarbaldehyde | 626-19-7 | 0.183 | 0.106 | -0.094 | -0.178 | 134.14 | 1.40 ^c^ | 0 | 0 |
| 554 | 2,5-dihydroxybenzaldehyde | 1194-98-5 | 0.277 | 0.063 | -0.068 | -0.157 | 138.13 | 0.54 ^m^ | 0 | 2 |
| 555 | 2-hydroxy-benzaldehyde | 90-02-8 | 0.424 | 0.194 | -0.067 | -0.180 | 122.13 | 1.81 ^m^ | 0 | 1 |
| 556 | 3-bromo-4-hydroxybenzaldehyde | 2973-78-6 | 0.610 | 0.722 | -0.071 | -0.179 | 201.02 | 1.83 ^m^ | 1 | 1 |
| 557 | 3-ethoxy-2-hydroxybenzaldehyde | 492-88-6 | 0.850 | 0.313 | -0.060 | -0.168 | 166.19 | 1.53 ^c^ | 0 | 1 |
| 558 | 4-hydroxynaphthalene-1-carbaldehyde | 7770-45-8 | 1.050 | 0.799 | -0.072 | -0.152 | 172.19 | 2.44 ^c^ | 0 | 1 |
| 559 | 4-(phenoxy)benzaldehyde | 67-36-7 | 1.257 | 0.940 | -0.062 | -0.182 | 198.23 | 3.15 ^c^ | 0 | 0 |
| 560 | 2-nitrobenzamide | 610-15-1 | -0.720 | 0.054 | -0.106 | -0.163 | 166.15 | -0.15 ^m^ | 0 | 1 |
| 561 | 3-nitroaniline | 99-09-2 | 0.030 | 0.331 | -0.089 | -0.143 | 138.14 | 1.37 ^m^ | 0 | 1 |
| 562 | 2-(4-nitrophenyl)acetonitrile | 555-21-5 | 0.130 | 0.400 | -0.104 | -0.192 | 162.16 | 1.37 ^m^ | 0 | 0 |
| 563 | 1-fluoro-2-nitrobenzene | 1493-27-2 | 0.250 | 0.461 | -0.098 | -0.186 | 141.11 | 1.69 ^m^ | 1 | 0 |
| 564 | 1-bromo-4-nitrobenzene | 586-78-7 | 0.380 | 1.087 | -0.105 | -0.174 | 202.01 | 2.55 ^m^ | 1 | 0 |
| 565 | 4,5-dimethyl-2-nitroaniline | 6972-71-0 | 0.450 | 0.693 | -0.081 | -0.140 | 166.20 | 2.07 ^m^ | 0 | 1 |
| 566 | 1-methoxy-4-nitrobenzene | 100-17-4 | 0.540 | 0.369 | -0.086 | -0.170 | 153.15 | 2.03 ^m^ | 0 | 0 |
| 567 | ethyl-4-nitrobenzoate | 99-77-4 | 0.710 | 0.927 | -0.109 | -0.183 | 195.19 | 2.33 ^m^ | 0 | 0 |
| 568 | 1-ethyl-4-nitrobenzene | 100-12-9 | 0.800 | 0.713 | -0.092 | -0.186 | 151.18 | 3.03 ^m^ | 0 | 0 |
| 569 | 1-methyl-2,4-dinitrobenzene | 121-14-2 | 0.870 | 0.813 | -0.115 | -0.189 | 182.15 | 1.98 ^m^ | 0 | 0 |
| 570 | 1-bromo-3-nitrobenzene | 585-79-5 | 1.030 | 1.102 | -0.106 | -0.169 | 202.01 | 2.64 ^m^ | 1 | 0 |
| 571 | 4-nitronaphthalen-1-amine | 776-34-1 | 1.120 | 0.690 | -0.084 | -0.139 | 188.20 | 1.77 ^m^ | 0 | 1 |
| 572 | 1,2-dinitrobenzene | 528-29-0 | 1.250 | 0.650 | -0.117 | -0.182 | 168.12 | 1.69 ^m^ | 0 | 0 |
| 573 | 1,3,5-trichloro-2-nitrobenzene | 18708-70-8 | 1.430 | 1.577 | -0.096 | -0.190 | 226.44 | 3.69 ^m^ | 3 | 0 |
| 574 | 5-fluoro-2,4-dinitroaniline | 367-81-7 | 1.690 | 0.887 | -0.113 | -0.155 | 201.13 | 1.31 ^c^ | 1 | 1 |
| 575 | 1-chloro-2,4-dinitrobenzene | 97-00-7 | 1.980 | 1.237 | -0.125 | -0.181 | 202.56 | 2.17 ^m^ | 1 | 0 |
| 576 | 1,5-dichloro-2,3-dinitrobenzene | 28689-08-9 | 2.420 | 1.660 | -0.130 | -0.167 | 237.00 | 2.99 ^c^ | 2 | 0 |
| 577 | 3-cyano-4,6-dimethyl-2-hydroxypyridine | 769-28-8 | -0.700 | 0.372 | -0.064 | -0.191 | 148.18 | 2.13 ^c^ | 0 | 1 |
| 578 | phthalonitrile | 91-15-6 | -0.340 | -0.041 | -0.095 | -0.199 | 128.14 | 0.99 ^m^ | 0 | 0 |
| 579 | methyl-4-cyanobenzoate | 1129-35-7 | -0.060 | 0.317 | -0.089 | -0.194 | 161.17 | 1.64 ^c^ | 0 | 0 |
| 580 | 4-bromobenzonitrile | 623-00-7 | 0.290 | 0.697 | -0.072 | -0.198 | 182.02 | 2.42 ^m^ | 1 | 0 |
| 581 | 3,5-dinitrobenzonitrile | 4110-35-4 | 1.220 | 1.139 | -0.140 | -0.186 | 193.13 | 1.82 ^c^ | 0 | 0 |

^a^ From the SVR model.

^b^ m indicates a measured logP, and c indicates a calculated logP.
